# How to Completely Squeeze a Fungus—Advanced Genome Mining Tools for Novel Bioactive Substances

**DOI:** 10.3390/pharmaceutics14091837

**Published:** 2022-08-31

**Authors:** Andreas Schüller, Lena Studt-Reinhold, Joseph Strauss

**Affiliations:** Institute of Microbial Genetics, Department of Applied Genetics and Cell Biology, University of Natural Resources and Life Sciences Vienna, A-3430 Tulln/Donau, Austria

**Keywords:** genome mining, secondary metabolism, BGC prediction, BGC activation, OSMAC, chromatin, CRISPR/Cas, dCas, cluster activation, filamentous fungi

## Abstract

Fungal species have the capability of producing an overwhelming diversity of bioactive substances that can have beneficial but also detrimental effects on human health. These so-called secondary metabolites naturally serve as antimicrobial “weapon systems”, signaling molecules or developmental effectors for fungi and hence are produced only under very specific environmental conditions or stages in their life cycle. However, as these complex conditions are difficult or even impossible to mimic in laboratory settings, only a small fraction of the true chemical diversity of fungi is known so far. This also implies that a large space for potentially new pharmaceuticals remains unexplored. We here present an overview on current developments in advanced methods that can be used to explore this chemical space. We focus on genetic and genomic methods, how to detect genes that harbor the blueprints for the production of these compounds (i.e., biosynthetic gene clusters, BGCs), and ways to activate these silent chromosomal regions. We provide an in-depth view of the chromatin-level regulation of BGCs and of the potential to use the CRISPR/Cas technology as an activation tool.

## 1. Introduction

### 1.1. Fungal Secondary Metabolites as Biopharmaceuticals of Tomorrow

Bacteria, plants, and filamentous fungi are capable of producing many chemically diverse bioactive substances, referred to as natural products or secondary metabolites (SMs) [1]. Probably the most prominent of these fungal bioactive compounds is penicillin from *Penicillium rubens*, discovered by Alexander Fleming in 1929 [2,3] which initiated the “antibiotic revolution” that lead to a major paradigm shift in the treatment of patients [4]. Many fungi and their SMs made it into pharmaceutical application, such as cyclosporin from *Tolypocladium inflatum* which is used as an immunosuppressant [5,6], or lovastatin as a treatment for coronary heart diseases produced by *Aspergillus terreus* [7]. Other fields of application for microbial-derived natural products are the food industry (e.g., soy sauce, sake, and miso by *Aspergillus oryzae* or citric acid by *Aspergillus niger* [8]), the agricultural sector (biocontrol agents [9,10,11], growth hormones [12]) and many others [13,14,15,16]. The ever-rising demand due to the emergence of multi-resistant pathogens and resistant cancer types underscores the importance of mining new bioactive substances from fungal sources [17].

SMs are per definition not essential for growth but may provide selective advantages for the producing organism upon specific environmental challenges: e.g., pH stress (acids and alkaline substances [18]), temperature stress (regulators of membrane fluidity [19]), oxidative stress (antioxidants [20]), UV protection (pigments [21]), microbial warfare (antimicrobials [22]), communication (e.g., quorum sensing via homoserine lactones [23]), pathogenicity and host-infection (effectors or virulence factors [24,25]), support of ion uptake (sideophore production for iron acquisition [26]). As versatile as these functions are, as diverse are the chemical properties of such natural products. Not surprisingly, most of them are only produced under defined environmental or developmental conditions implying a complex regulation at the molecular level [27].

The blueprints for such bioactive substances are stored in biosynthetic genes that are, in most cases, physically linked in fungal genomes and together with genes for regulators and transporters sometimes constitute very large BGCs [28,29]. These BGCs are silenced by default through heterochromatic structures at the chromosomal level, but once activated by the specific signal, they work similar to an orchestrated factory to produce the cluster-specific compound. Bioinformatic tools have been developed that predict fungal BGCs based on whole-genome sequence data and these predictions show that so far sequenced fungi harbor between ~15 and 50 BGCs, but up to more than 80 BGCs have also been predicted in some genomes [28,30]. However, as outlined above, most of them are transcriptionally silent under standard laboratory conditions and hence the majority of associated natural products are not known [27]. 

### 1.2. Genomic Organization and Evolution of BGCs

Depending on the so-called backbone or key enzyme(s) involved in the biosynthesis, SMs are categorized according to the chemical families. They belong to e.g., polyketides, non-ribosomal peptides, terpenes and alkaloids or prenylated tryptophan derivates. Accordingly, they are synthesized by polyketide synthases (PKSs; further divided into highly reducing (HR-PKS) and non-reducing (NR-PKS)), non-ribosomal peptide synthetases (NRPSs), terpene cyclases (TCs) and dimethylallyltryptophan synthases (DMATSs) or hybrids of them [28,29]. 

Next to the backbone enzyme-encoding gene(s) BGCs often harbor genes involved in self-resistance against a potentially toxic SM produced by the BGC, transport of precursors, intermediates, or the resulting compound [31,32], transcriptional activation of the biosynthetic genes, and often so-called tailoring or decorating enzymes that modify the structure of the chemical scaffold compound released by the backbone enzyme (e.g., P450 monooxygenases, methyltransferases, hydroxylases, epimerases, etc. [29]). 

A few exceptions exist that deviate from this standard configuration. For example, in *Fusarium graminearum*, polyketide synthase PKS8 was shown to be a stand-alone gene that is responsible for the synthesis of gibepyrone A [33], while the kojic acid BGC from *Aspergillus oryzae* does not have a backbone enzyme-encoding gene at all [34]. In other cases, the formation of superclusters was observed which encode for several independently synthesized biosynthetic products [35]. The opposite was also observed, where several BGCs or genes from distinct genomic locations communicate with each other to synthesize one product [36].

Fungi exhibit a very diversified and specialized chemical repertoire. The diversification of BGCs increases with the phylogenetic distance [37,38] but in some cases, it can even be observed within the same or closely related species, which was shown for *Aspergillus fumigatus* [39,40], *Aspergillus niger* [41] as well as between *A. oryzae* and its close relative *Aspergillus flavus* [42]. Constant interactions with other microorganisms and environmental conditions promote the intra and interspecies diversification process by exerting positive or negative selective pressure on BGCs in that specific ecosystem. If a BGC is non-essential or not beneficial in a certain microenvironment it is likely to acquire deleterious mutations or even gene loss over time [43]. A comparison of isolates from the same species but separated microenvironments revealed the emergence of different ecotypes as observed during population genetics studies on *Streptomyces* [44] and *Umbilicaria pustulata* [45].

BGCs are differently affected by evolution compared to the rest of the genome. They are located outside of so-called syntenic blocks, i.e., their position within the genome, and the BGC itself is less conserved than the rest of the genome [46,47]. In support of this, many BGCs were found to be enriched in sub-telomeric regions which are known to be hotspots for recombination [48]. This was shown in *Sclerotinia sclerotium* [49], *Magnaporthe oryzae* [50,51], *A. fumigatus* [52], and *Zymoseptoria tritici* [53].

From an evolutionary perspective, the ability to quickly adapt to new environmental challenges is fundamental for the survival of an organism. In the case of fungi and other microbes, this means that they can adapt and change their metabolomic arsenal fast enough to properly compete against other microbes or environmental changes. This implies that the relevant genomic regions and hence the genes situated in these regions (i.e., BGCs) experience regular mutations (single nucleotide polymorphisms (SNPs) such as missense, nonsense, frameshift, or gain/loss of function) and genomic rearrangements as for instance gene duplications, gene or BGC loss, BGC rearrangements, pseudogenization events and also the acquisition of genetic material via horizontal gene transfer [43]. Organisms that acquire beneficial mutations will surpass others that experience disadvantageous ones whereby the environmental context decides if a certain mutation is beneficial or not. Microbial competition is not only about having the “best weaponry” but also about defense, resource efficiency, and denying beneficial effects to competitors. This not only implies gain of function or the *de novo* assembly of a BGC but also reductive genomic evolution which led to the “Black Queen Hypothesis” (BQH) [54]. The BQH considers that selective pressure can lead to the loss of a gene or function if that gene is dispensable (which makes it more energy efficient not to have it), or if the gene product would give a competitor more benefits than the producing organism itself.

For the artificial activation of silent BGCs in a laboratory context, this means that some of the BGCs will carry deleterious mutations or even miss whole genes that would be necessary for the biosynthesis of the BGC product. In these cases, solely transcriptional activation will not suffice. Close examination of the BGC of interest can already unveil if genes carry a premature stop codon or if homologous BGCs indicate missing genes or frameshift mutations. A possible workaround in these scenarios is the exchange of single genes by their homologs or the genetic repair of a nonsense mutation as performed by Burkhardt et al. with the STC5 gene in *Fusarium fujikuroi* [55].

Multiple genomic, inter-species, and environmental factors contribute to this elevated diversification and evolution of BGCs. The occurrence of mutations and genomic rearrangements depend on many exogenous (e.g., radiation, heat, chemicals, environmental conditions, …) and endogenous factors (e.g., errors in DNA damage repair, or active transposable elements). As stated above, BGCs and pathogenicity-related genes are often in polymorphic regions that are prone to such events (e.g., sub-telomeric regions). Transposable elements (TEs) play an important role during evolution and also in the de novo assembly of BGCs. In *Verticillium dahliae* it was found that BGC and pathogenicity-related genes are often located close to transposable elements [56]. In a comparative study within *Aspergillus fumigatus* isolates, horizontally transferred BGCs, BGC idiomorphs (i.e., different BGCs in the same genomic location), and BGCs that are present in different genomic locations within the same or closely related species) are often flanked by TEs [39]. Similar findings were also found between *A. oryzae* and *A flavus* where two strain isolates had different BGCs in the same genomic locus [42] or in the case of several *Botrytis* species where the BOT BGC, involved in botrydial biosynthesis, was present at four different genomic locations across seven examined species [57,58]. When analyzing BGC genes and TE co-occurrence in *Sclerotinia,* they found that the overall co-occurrence of BGC genes and TEs is not significantly enhanced. Upon closer examination, however, it became evident that a subset of BGC genes, which is enriched in key-enzyme encoding genes, is co-occurrent with TEs and that genes in this subset often derive from recent gene duplication events [49]. BGC genes near TEs, therefore, were found less conserved than BGC genes more distal to the TE. All these findings point towards a TE-dependent heterogeneous evolution of BGCs (i.e., genes near TEs have an increased “evolutionary speed”). In the case of BGC prediction, TEs are, to our knowledge, not yet a considered factor, although it seems that these motives could very likely be good indicators for the presence of a BGC. Furthermore, TEs are probably also important factors for the de novo assembly of BGCs. 

De novo assembly is hypothesized to be a two-step process. The first step starts with the framework establishment of the new metabolic pathway [59]. Often, this is accomplished by horizontal gene transfer, as shown for the sterigmatocystin cluster from *A. nidulans* to *Podospora* [60], or the transfer of the bikaverin BGC from *Fusarium* to *Botrytis* [61] and many others [59,62,63,64,65,66]). Other mechanisms involve the recruitment of host genes or gene duplication events (e.g., fatty acid synthase as ancestor of PKS enzymes [67]). In a second step, the BGC is clustered by genomic rearrangements. Sometimes, however, it also happens that two separate BGCs, which are not physically linked, are responsible for the biosynthesis of one compound (i.e., cluster crosstalk) [36] or that superclusters (e.g., fumagillin/pseurotin cluster from *Aspergillus fumigatus* [35]) are formed. Cluster crosstalk could be observed with the trichothecene supercluster of *F. graminearum* which consists of a 12 gene BGC and a separated 2 gene BGC plus one single gene at three different genomic loci [68].

All in all, the genomic comparative studies from *A. fumigatus* [39], *A. flavus* and *A. oryzae* [42], *S. sclerotiorum* [49], *V. dahlia* [56], and also the reviews from Lind et al. [39] and Rokas et al. [43] give very good insights into the evolution of BGCs and especially the role of TEs as one of their driving forces.

The fact that this evolutionarily driven arms-race is not only apparent between closely related species but also between different ecotypes of the same species illustrates that BGCs are evolving at a fast pace. This highlights that the research pool of fungal genera and species must be expanded to increase the chance of finding novel natural products in the future. However, most of the current research is focused on mining BGCs in only a few but very well-described genera, the so-called model organisms.

In this review, we highlight the concepts and technical possibilities on how to decrypt the secondary metabolome of a given (filamentous) fungus by efficiently deploying targeted and untargeted BGC-activation methods in synergy with genome mining. Special attention will be paid to more recent strategies involving the chromatin regulation of BGCs and the new possibilities currently emerging by the combination of CRISPR/Cas technology and transcriptional activators, global regulators, or chromatin-modifying enzymes.

## 2. Deciphering the Genomic Potential of Fungal Genomes for Secondary Metabolites 

In silico-prediction of BGCs within the genome of the target fungus gives important information about the (theoretical) chemical potential of a fungus. This is not only important for deciding if a species is a promising candidate for novel bioactive compounds but is also a prerequisite for linking those compounds to a specific cluster and also for targeted BGC activation. Bioinformatic analyses of high-quality genome sequences and transcriptome datasets are becoming cornerstones to explore the theoretical chemical diversity of SMs in a given species. Those resources are routinely used today for gene annotation [69], BGC prediction [70], cluster phylogeny [28,71,72], prediction of the metabolite structure and bioactivity [73], drug target predictions [74,75] and also dereplication [76]. As outlined in Section 1.2, there are many different data types and sources that can be utilized for the prediction of BGCs. In the following paragraph, we will describe the basic principle and the developmental cycle of BGC prediction tools. 

**Figure 1:**
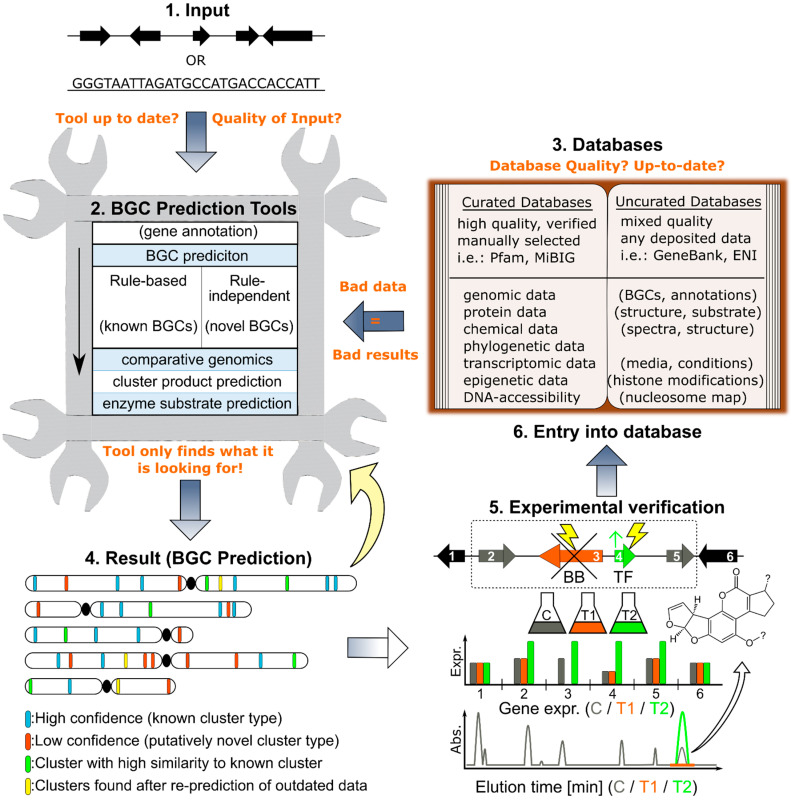
Workflow for genome mining assisted BGC prediction and verification. The order and interplay of prediction tools, databases, and scientist is depicted. In orange letters, we point out pitfalls that could lead to incorrect predictions and can easily be overlooked. The correct order is depicted by the arrows and follows the numbered bold headlines. The light-yellow arrow represents a re-prediction of an outdated BGC annotation. Explanations regarding point 5. “Experimental verification”: The top part is the genomic locus with the putative BGC surrounded by a dotted outline; genes are numbered from 1–6, BB stands for “backbone- gene” (gene No. 3; orange) and TF stands for “pathway-specific transcription factor” (gene No. 4, green). Yellow lightning bolt symbols represent genetic manipulation (i.e., gene deletion in case of BB or overexpression in case of TF). The colors represent the three different samples/transformants: The control (“C”) represents the wildtype (wt) or recipient strain and is illustrated by a grey flask, expression bars, and HPLC spectra (i.e., wt-like synthesis of the investigated secondary metabolite. In this case it is produced but in case of a silent BGC, the peak on the far right would be absent). Transformant 1 (“T1”) has the backbone gene deleted and is represented by an orange flask, gene expression bars and HPLC peaks (orange flat line; i.e., no synthesis of the secondary metabolite); Transformant 2 (“T2”) has the TF of the BGC overexpressed. It is depicted by a green flask, green gene expression bars and a green HPLC peak (i.e., overproduction of the secondary metabolite). Diagrams have either the expression level (“Expr.”) or the absorption value (“Abs.”) as the y-axis and time (“min”) as the x-axis. The setup of 5. Experimental verification represents one possibility of linking a putative BGC to its metabolite. After deposition into a database (6.), the gathered information about the BGC can be utilized for the prediction of BGCs in other genomes (2.).

Figure 1 illustrates the workflow of genome mining-assisted BGC prediction and revelation. It comprises the crucial steps that are necessary for the prediction and verification of a BGC and its corresponding metabolite. It also shows the pitfalls which can easily remain unnoticed but significantly impact the reliability of the prediction.

### BGC Prediction Methods

The majority of BGC prediction tools can be classified into either high-confidence/low-novelty (rule-based) or low-confidence/high-novelty (rule-independent) [70,77]. 

High-confidence/low novelty methods reliably detect BGCs that are closely related to already verified cluster types (i.e., PKS; NRPS, etc.) by scanning the genome for their specific signatures (genes, gene types, genomic arrangement, etc.). These signatures are retrieved from experimentally verified data that links the compound to the respective BGC, which are then implemented into the algorithm. The prediction tool antiSMASH [78] is a very well-established and regularly updated platform that also integrates many other published algorithms. It can be used for fungal, plant, and bacterial genomes and contains methods for finding canonical and also novel BGCs. During the prediction of known BGC types, the procedure often starts with an initial screen for backbone enzyme-encoding genes which serve as anchor-point for further analysis of the region. In a second step, the cluster borders are determined by scanning the area around this anchor point for the presence of other BGC-related genes [78,79]. This means, however, that high-confidence/low-novelty (rule-based) methods can only detect BGCs that share similarities with already discovered and described cluster types

In contrast to this, low-confidence/high-novelty (rule-independent methods) pursue the aim of detecting BGCs of a novel yet unknown type that does not follow currently known patterns (gene types, arrangement, etc.). There are multiple strategies for finding new BGC types that use various experimental and -omics data for this purpose. 

MIPS-CG, for example, utilizes an algorithm that does not rely on the use of backbone genes as BGC-anchor points as some BGCs were shown to lack these (e.g., kojic acid BGC). Instead, to identify these non-canonical BGCs, MIPS-CG performs pairwise genome comparisons between different fungal species and removes syntenic blocks, because BGCs are often not located in such regions. Following the assumption that BGCs follow a basic common structure (similar gene functions) MIPS-CG scans for clustered genes that follow this pattern [47]. 

The EvoMining algorithm uses a phylogenomic approach to detect BGCs. It is based on three evolutionary concepts. First, those new enzymatic functions evolve by retaining the reaction mechanism of the enzyme but changing substrate specificity. Second, the evolution of metabolic pathways can lead to the deployment of existing enzyme families that perform new functions (e.g., fatty acid synthases as the ancestor of PKSs) and third, that BGCs have their origin in central metabolism [71,80].

AntiSMASH version 4 introduced the Clusterfinder algorithm which converts a genome into a string of Pfam domains (i.e., protein family domains). Subsequently, BGCs are annotated where stretches with a significant amount of BGC-related Pfam domains occur [81,82]. 

The ARTS algorithm (Antibiotic Resistant Target Seeker), although currently supporting only bacterial genomes, is a tool for specifically finding BGCs that encode for an antibiotic. When bacteria produce an antibiotic substance, they can either detoxify and shuttle the compound out of the cell or express a resistant homolog of the gene that is targeted by the antibiotic. The resistance pathway is exploited by ARTS. The algorithm compares the genome to a reference genome and looks for duplicate housekeeping genes in the genome. These resistant homologs often are inside the same BGC. Furthermore, the cluster is often acquired by horizontal gene transfer (HGT). With the combination of BGC prediction via antiSMASH, gene duplicate screening and the comparison of the phylogeny of core gene trees of the organism and the cluster genes, ARTS can predict clusters that are highly likely to be responsible for the biosynthesis of an antibiotic [83,84]. The FRIGG pipeline pursues a similar approach but can be used for fungal genomes [85]. 

Middas-M follows the hypothesis that BGCs can be found where an array of genes is coregulated. They combine transcriptomic data of several culture conditions with genomic data to predict BGCs [86]. This method will, however, only catch BGCs that actually can be activated under laboratory conditions. 

Algorithms use various curated but also non-curated data sources for their calculation of BGC genes. These data repositories can basically hold any kind of information about BGCs (e.g., gene information, genomic arrangement, related species, protein information, educts and products, transcriptional information, phylogenetics, environmental information, etc.). Curated data sources contain fewer data entries, but the reliability and quality of these entries are often pristine, non-redundant, and experimentally backed which leads to more reliable predictions. Non-curated data sources basically hold all the information that is available (e.g., Genbank). This means that the algorithm has to process and filter a lot of data of various quality and actuality (e.g., low-quality sequencing data and annotation, experimentally not verified predictions, etc.). Depending on the popularity of the examined genus this also can lead to many redundancies that might contain flawed information. All in all, non-curated sources contain more, but less reliable data which can reduce the quality and reliability of the prediction.

It should be mentioned that, as the experimental information about a novel BGC type increases, it will subsequently pass over from the rule-independent (high novelty/low confidence) to the rule-based (low novelty/ high confidence) prediction methods. This developmental process of prediction tools will undoubtedly lead to the discovery of yet unknown BGCs in already known and well-described species; however, it is strongly dependent on high-quality and accurate experimental data, provided by laboratories. The elucidation of novel BGC types and their genomic signatures, plus their proper deposit in data repositories (e.g., MiBiG, NP-atlas 2.0, GNPS, etc.) will significantly improve BGC predictions in the future. For more detailed information about genome mining and predictive tools, please consult the review of Blin and coworkers from 2019 [70].

Table 1 lists many of the actual databases and BGC prediction tools. They are ordered according to their last update (Status: March 2022), and also summarizes their characteristics.

## 3. Activation of Microbial BGCs via Environmental Triggers

In the past decades, many strategies have been developed to shed light on this “dark matter” in fungal genomes and thereby unlock the chemical wealth residing within the fungal kingdom. In the pre-genomics era approaches predominantly sought to induce SM biosynthesis by mimicking environmental triggers. Genome sequencing and bioinformatic analyses for BGC predictions were developed later on and did not only reveal that filamentous fungi possess far more BGCs than anticipated, but also opened opportunities for a more targeted activation of single BGCs. 

We are now at a point where the combination of technological possibilities, abundance of available methods, and the already substantial amount of collected data makes it possible to expand the research to novel yet not well described fungal species. There is no “one for all”-approach that is superior to other strategies, but available methods should rather be used in a complementary and context-dependent manner.

### 3.1. Mimicking Natural Habitats and Stress Conditions

Fungal SMs serve different purposes during the life cycle of a fungal colony and accordingly, their production profiles somehow mirror these environmental or developmental conditions. Therefore, one of the approaches to trigger the production of new SMs is to impose challenging, sometimes stressful growth conditions on the fungal cells. Indeed, testing different cultivation conditions and imposing environmental stress has proven to be a very effective way of triggering the production of otherwise absent secondary metabolites. This approach was termed “One Strain-Many Compounds” (OSMAC) [115,116] and is very useful for assessing which BGCs can be induced within a laboratory environment and which need more laborsome methods such as genetic manipulation for their activation. Especially in the case of less-studied fungi that are in the process of being established for laboratory work, different cultivation conditions also give insights into the fungal physiology and suitable media and parameters for their cultivation. 

In some instances, it was shown that the fungus reacted as expected to the applied conditions. For example, UV light exposure can trigger the production of UV protectants [117], while oxidative stress can lead to the production of antioxidants [118]. It was also shown that *A. terreus* produces the pathogenesis-related SM terrain when fungal culture conditions mimic the environment of the plant or rhizosphere, which is associated with nitrogen starvation, iron shortage, and the presence of methionine [119]. 

Environmental stimuli are generally processed via global regulators. The purpose of global regulators is to coordinate the expression of an array of genes that ensure a resource-efficient response that is suited to the environmental circumstances [120,121]. Therefore, instead of mimicking such stimuli via media and cultivation conditions, the genetic engineering of global regulators (i.e., deletion, overexpression, knockdown, mutation) is also a successful way of triggering the activation of BGCs. This, however, can also lead to phenotypical abnormalities such as aberrant sexual and asexual cycle and growth defects [122,123] which could be linked to the intertwined character of many global regulatory networks [29]. 

The most thoroughly researched environmental signals that are connected to the production of fungal metabolites are nutrient availability and light regulation. 

The type and concentration of the available nitrogen source also greatly impacts secondary metabolism, as shown for some well-established model organisms from the genus *Fusarium* [124,125,126,127] and *Aspergillus* [128]. The major GATA transcription factor AreA, AreB, and Nmr are global regulators of nitrogen-dependent genes and pathways in filamentous fungi. In *F. fujikuroi*, deletion of *AREA* and *AREB* led to differential regulation of many known but also yet cryptic BGCs [127]. In *F. graminearum*, AreA is required for the production of deoxynivalenol (DON), zearalenone, and fusarielin H [129]. Similar results were obtained in *Acremonium chrysogenum* where the deletion of *areA* led to the reduction of cephalosporin [130] and in *Fusarium oxysporum* where AreA is necessary for the synthesis of beauvericin and ferricrocin [131].

Light is also an important natural regulator of growth, development/conidiation and biosynthesis of natural products in fungi [132,133,134,135,136]. Genetic manipulation of components of the fungal-specific light regulator velvet complex showed its involvement in many developmental and also biosynthetic processes [137,138,139,140,141]. It consists of velvet-like proteins like, VelA, VelB and most importantly, the methyltransferase LaeA. Deletion of *laeA* in *A. nidulans*, resulted in the abolishment of the production of sterigmatocystin, penicillin, and lovastatin while overexpression of *laeA* led to increased lovastatin and penicillin production [142]. Similarly, LaeA was also found to be a positive regulator of secondary metabolism in *Valsa mali* [143], *Aspergillus ochraceus* [136], *Penicillium expansum* [144], *Chaetomium globosum* [145], *Fusarium verticillioides* [146] *and F. fujikuroi* [147]. Therefore, LaeA seems to have a conserved role of a positive regulator of BGCs in many filamentous fungi which makes it a promising target for overexpression studies. 

Other variables influencing SM levels are the pH value of the growth medium [148,149,150,151,152,153], an effect mainly mediated by the transcription factor (TF) PacC [154,155,156], temperature stress [118,157,158], osmotic stress, and salinity [159,160,161,162], or metals and trace elements [163,164,165,166]. The concentration and quality of the available carbon source can also be a critical parameter [135,167,168] for SM production which is mediated by the carbon catabolite repressor CreA [122]. For oxidative stress [118,123,169,170,171] Skn7 [172] and ApyapA/AoyapA (Aflatoxin production) are known response regulators [173,174]. There are general repressors such as McrA, but the conditions for its activity remain unknown [175] or Nsd1(CsM1/Ltf1) which is involved in sexual development and conidiation in *A. nidulans* and other fungi [176]. 

Apart from media composition, the type of cultivation condition also can play a pivotal role in the physiology of the fungus and thus potentially influence the production of secondary metabolites. Differences in the metabolomic profiles could be observed when comparing submerse and solid cultivation [177,178], as well as between agitated or static fermentation [179]. The addition of a polymeric growth support to a submersed culture also had profound influences on growth and metabolite biosynthesis [180].

Table 2 provides an overview on methods and examples where they have been used to successfully induce BGCs by mimicking natural triggers or by genetic manipulation of global regulators.

### 3.2. Co-Cultivation

During co-cultivation [116,211,212], microorganisms are brought into an interactive environment with the aim to mimic natural competition that can also trigger the production of SMs. There are several possible setups for co-cultivation. One approach uses (random) pairwise co-cultivation of strains [213] (Table 3; Setup 1). A second approach tests a strain collection against certain microorganisms of interest, such as (antibiotic-resistant) pathogens [214,215] (Table 3; Setup 2). A third approach cultivates strains together with the (sterile-filtrated or extracted) culture supernatants of other microorganisms. The latter approach can be necessary if cultivation or media requirements of the individual species are not compatible for simultaneous cultivation or if the mechanisms of the induction of SMs (e.g., physical interaction or presence of a compound) is being investigated [216] (Table 3; Setup 3). This latter strategy was just recently chosen in our laboratory resulting in the identification of the signaling molecule, that triggers the fungal response in the interaction between the bacterium *Streptomyces rapamycinicus* and the filamentous ascomycete *A. nidulans* [184]. Another fruitful concept is the co-cultivation of a fungus with a bacteriophage (i.e., infection) which led to the production of a novel SM [217]. Co-cultivation can be very challenging due to the sheer endless combinatorial numbers of specimens and cultivation parameters that have to fit both organisms. However, if the co-cultivation approach is already well established, it can be a very powerful and rewarding tool. Table 3 lists publications that successfully applied co-cultivation approaches for the activation of BGCs. 

### 3.3. Conclusions of Initial Assessment

OSMAC approaches, paired with metabolome analysis, transcriptome analysis, and bioinformatic methods can already create an extensive map of the secondary metabolome that is produced in a laboratory environment. To discriminate already known compounds from putatively new compounds, exact masses of the new compounds are compared to masses from already described compounds. This can be performed in the lab by screening against a library of SM standards [234] or by applying bioinformatic tools such as PRISM 4 [235] or DEREPLICATOR+ [76]. 

After this initial assessment, the remaining cryptic BGCs can then be targeted by molecular biology methods for releasing the full bioactive potential of the target organism.

## 4. BGC Activation Using Molecular Biology Tools: The Way to Fully Exploit the Genome for Novel Bioactive Substances 

**Figure 2:**
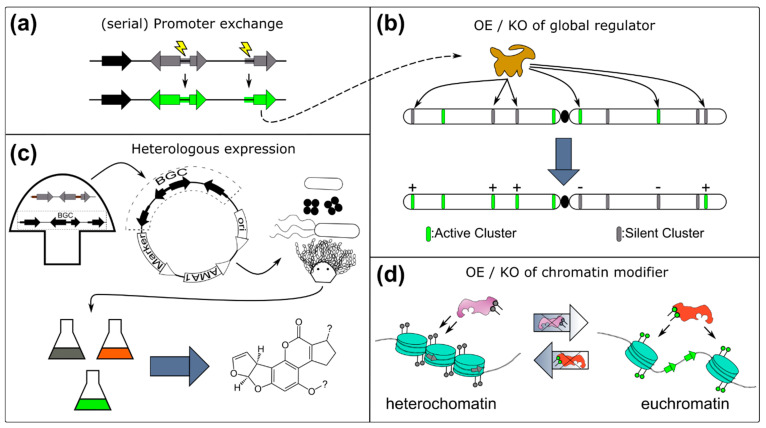
Molecular biology methods for BGC activation. Silent genes are depicted as grey or black arrows while active genes are green. Yellow thunder bolts represent genetic manipulation. Genetic manipulation that results in overexpression or deletion of a gene is abbreviated as “OE” or “KO” respectively. (**a**) The exchange of one (i.e., pathway-specific TF) or several promoter regions of the BGC of interest leads to gene activation. (**b**) Overexpression of a global regulator can lead to the activation of several BGCs all over the genome. (**c**) Heterologous expression of a BGC of interest in an expression host followed by cultivation, extraction and structure elucidation of the compound encoded within the BGC. (**d**) Genetic manipulation of a chromatin remodeler can lead to the de-repression or silencing of a BGC that is regulated by the respective remodeler. Silencing and expression-promoting histone modifications are depicted as grey and green pins respectively.

Applying molecular biology tools for genetic engineering enables the alteration of regulatory mechanisms at the genomic or epigenetic layer. This way, cryptic BGCs not transcribed under any tested laboratory condition can be activated in the native host by the manipulation of global regulators, pathway-specific regulators, chromatin modifiers, or by disruption of a repressive signaling pathway. Furthermore, if the fungus is not amenable to any approach that requires genetic transformation, the whole BGC may be transferred to and expressed in another organism (i.e., heterologous expression). There are untargeted methods that result in a global metabolomic and transcriptional response (e.g., deletion of global repressor or overexpression of global activator) and there are targeted methods, that aim to activate only a specific BGC by manipulating regulatory elements of the BGC (e.g., overexpression of pathway-specific transcription factor (TF)). The advantage of targeted methods is that changes in the metabolome can be much more easily linked to the BGC under study. Figure 2 illustrates the molecular genetic methods for the activation of silent BGCs. 

### 4.1. Overexpression of a Pathway Specific Transcription Factor or Whole BGC

By overexpression of a pathway-specific TF, the whole cluster may be upregulated. This strategy was shown to be successful in increasing the production of aflatoxin in *A. flavus* [236] and also led to the discovery of aspyridone [237], and asperfuranone in *A. nidulans* [238] or trichosetin in *F. fujikuroi* [239]. Further examples can be retrieved from Table 4.

Bioinformatic analyses revealed that in the case of the genus *Aspergillus* 50% to up to 70% of the BGCs contain a pathway-specific TF [240,241]. Co-expression studies under different conditions in *Aspergillus niger,* however, provided evidence that eight of 45 BGCs that contain a TF, show no co-expression between the BGC-synthetic genes and the TF, which indicates that they are not affiliated with the BGC and hence, overexpression would not lead to its activation [242]. This hypothesis is supported by a publication where overexpression of the pathway-specific TF failed in 15 of 18 cases in *A. nidulans* [243]. This approach also fails if the TF needs to get post-translationally activated, as it is the case with AflR, the pathway-specific TF of the sterigmatocystin cluster of *A. nidulans,* which is inactivated by the presence of glucose [244] and activated by the RimO kinase in response to glucose starvation, as recently discovered in our laboratory [245]. Another possibility could be a necessary interaction between the BGC-specific TF and another factor or natural inducer to unfold the full activation potential. In this scenario, the overexpression of just the TF might not have any effect on the clustered genes [246]. A possible way to circumvent this was shown in the (+)-asperlin cluster in *A. nidulans*. The modular composition of many TFs (activator domain, DNA-binding domain) made it possible to fuse the DNA-binding domain of the asperlin BGC-specific TF (AlnR) with the trans-activator domain of another TF (afoA). This way, the overexpression of this hybrid TF led to the discovery of the antibiotic (+)-asperlin [246].

In the case of clusters that do not have a TF, serial promoter replacements of all BGC-specific genes can be conducted. This approach has been successful in the activation of several uncharacterized BGCs in *A. nidulans*, finally shown to be responsible for the expression of the Ivo gene cluster producing a new pigment [247], or for the discovery of the new proteasome inhibitor fellutamide B [248], and chicorine [243]. The idea was also tested in other species and Table 4 summarizes some of the successful attempts in the published literature. However, it is clear that exchanging many promoters in a multi-gene BGC can be quite cumbersome and may not always lead to successful transformation events, especially when the BGC under investigation is located in heterochromatic regions. 

### 4.2. Heterologous Expression

In the case that none of the aforementioned methods worked out or the studied species is not amenable to transformation or does not yield stable transformants, heterologous expression in a model host can be a powerful tool for the activation of unknown BGCs. For this method, single protein domains or genes or a whole BGC are introduced into a well-known production host. For the expression of single domains or genes, bacterial hosts such as *Escherichia coli* are often sufficient [272,273]. For larger genes or small clusters, many (engineered) yeast overexpression hosts such as *Saccharomyces cerevisiae*, *Hansenula polymorpha* or *Pichia pastoris* exist [274,275].

If the expression scaffold of the donor organism (promoters, terminators, codon usage, splicing, etc.) is however not suited for the host there are two other possibilities. The regulatory elements of a BGC can be exchanged by host-specific ones, or the BGC can be heterologously expressed in a host that is closely related to the donor organism [274]. There are already engineered fungal hosts (low metabolite background, removal of proteases) that are readily deployed such as *A. oryzae* [276], *A. nidulans* [277] *A. niger* [278], but also *F. grameniarum* [279,280] and *Penicillium* strains [281,282] are emerging as expression platforms. Heterologous expression is often used when high yields of a certain SM are important (i.e., industrial application) but specifically for the activation of silent fungal BGCs, systems such as FAC-MS [283,284], HeX [285] or CoIN [286] have been developed. Table 5 lists publications that used heterologous expression for the activation of silent BGCs.

#### 4.2.1. Fungal Artificial Chromosomes (FAC-MS) [283,284]

During the FAC-MS approach, the donors’ DNA is sheared and random chromosomal parts (up to 300 kb) which eventually contain a whole BGC are cloned into fungal artificial chromosomes (FACs). These FACs are then transformed into the host organism (e.g., *A nidulans*). This already led to the identification of 15 new metabolites from a library of 56 FACs each containing one unknown BGC from *A. aculeatus, A. terreus* or *A. wentii* in the heterologous expression host *A. nidulans.* It also resulted in the elucidation of the biosynthetic pathway of acu-dioxomorpholine from *A. aculeatus* [283,284,287]. As long as the expression host can utilize the AMA1-region and the donors’ genomic information, the FAC-MS approach is a viable option for BGC mining.

#### 4.2.2. CoIN [286]

This approach uses the *A. nidulans* regulatory matrix (TFs and their downstream promoters) of the sterigmatocystin cluster in combination with the *niiA*/*niAD* promoter. In this system, the TF-encoding genes *aflR* and its cofactor *aflS* are put under the control of the bi-directional promoter of the *niiA/niaD* genes coding for nitrate- and nitrite reductase, respectively, in *A. nidulans*. The genes of the BGC of interest are put under the control of the eight sterigmatocystin promoters that are regulated by AflR/AflS. This way, the orchestrated expression of up to eight genes of an unknown BGC without reusing a single promoter sequence can be accomplished by the growth of nitrate as the sole N-source leading to a high induction of the TFs.

#### 4.2.3. Single Promoter BGC Expression [296]

Hoefgen and coworkers utilized the 2A peptide of the picornavirus to enable the transcription and translation of a whole BGC with only one promoter and one terminator. The 2A peptide auto-cleaves itself after translation without leading to the disassembly of ribosomes. This way, several genes can be co-expressed with the use of only a single promoter. The cloning of the construct however can be difficult and laborious and the transcript levels of the genes will stay equal and cannot be individually regulated.

#### 4.2.4. Hex [285]

With the Hex approach, it is possible to express whole fungal BGCs in an engineered strain of *Saccharomyces cerevisiae*. New promoter sequences that are coregulated with p_ADH2_ (called Hex-promoters) have been utilized to enable cloning via a high throughput DNA assembly strategy by homologous recombination into 2 µ vectors. Genome mining assisted the selection of novel BCGs and subsequent cloning into yeast plasmids with an upper limit of seven genes per plasmid (maximum of two plasmids per clone). Expression is followed by untargeted metabolomics and LC-MS and NMR for structural elucidation of putatively novel compounds. For proof of concept, 41 investigated BGCs (from ascomycete and basidiomycete species) resulted in the detection of 22 new compounds.

## 5. Chromatin: An Important Layer of (BGC) Gene Regulation

While previously mentioned methods have already led to the discovery of many bioactive substances of fungal origin, many BGCs may still resist discovery with these approaches. Therefore, another strategy would be the manipulation of epigenetic and chromatin-based regulation of BGCs. Until recently, this could only be accomplished by the manipulation of global chromatin remodelers, however, as the general knowledge of this level of regulation increases, engineering of programmable chromatin regulators becomes feasible. The emergence of CRISPR/Cas-based methods, moreover, opens up new opportunities for activating BGCs by locus-specific modification of histones. These new approaches would not only make it possible to activate BGCs where other methods failed but have the potential of giving many new insights into the histone code of gene regulation. The next sections concentrate on the regulation of BGC expression via chromatin and histone modifications and describe also the current state of locus-specific chromatin modifications for the activation of secondary metabolites. After that, we will review the role of a CRISPR/Cas as a carrier for targeted BGC activation via locus-specific chromatin modification.

In eukaryotes, genomic DNA wraps around histone protein octamers (~146 bp DNA/nucleosome) to form nucleosome chains (i.e., chromatin) [298,299]. Each nucleosome consists of an octamer constituted of two of the four core histone proteins H2A, H2B, H3 and H4 with sometimes iso-forms (e.g., H2AZ, CENH3, H4.2, H4v) of the histones being incorporated into the nucleosome for the specific cellular processes (e.g., DNA repair, etc.) [300]. Histones, especially H3 and H4, can carry post-translational modifications (or histone marks) on specific amino acid residues, that are read and translated by so-called reader proteins. Depending on the histone marks, chromatin structure is either loosely packed and open for transcription or very compact which hinders access to the DNA and therefore transcription. Furthermore, chromatin reader proteins can also recruit other proteins such as TFs or components of the transcriptional machinery. This way, genes can be either packaged into a very tight polymer of nucleosomes that is transcriptionally silent, called heterochromatin, or into a more relaxed form of packaging, called euchromatin, that allows proteins to interact with the underlying DNA (i.e., TFs, transcriptional machinery, etc.) [301].

### 5.1. Heterochromatin

The DNA within heterochromatic regions is not freely accessible for transcriptional activation. Pioneering factors however are capable of binding to condensed chromatin and subsequently recruit TFs or chromatin remodelers. This way, heterochromatin can be reversed to a more open and accessible state which promotes the binding and interaction of TFs or other reader/writer/eraser proteins and subsequently can result in gene activation [302,303]. In general, there are two pathways to form heterochromatic regions within the genome which are conserved in most eukaryotes i.e., constitutive and facultative heterochromatin. Both chromatin states are associated with distinct histone methylation marks.

#### 5.1.1. Constitutive Heterochromatin via H3K9 Methylation and Clr4/HP1

Methylation of H3K9 is a hallmark of constitutive heterochromatin. H3K9me3 is established by the histone methyl-transferase KMT1 (ClrD in *A. nidulans, Schizosaccharomyces pombe* Clr4 homolog) [304] and the heterochromatin protein 1 (HP1, *A. nidulans* homolog HepA) which is a reader protein for this mark [305]. In some fungi, it was shown that the DNA methyltransferase (e.g., Dim-2 in *Neurospora crassa*) is subsequently recruited for further silencing of the region [306]. In the case of fungi, the term “constitutive” may be misleading as it was shown that *A. nidulans* can activate its sterigmatocystin cluster that contains hallmarks of constitutive heterochromatin (i.e., H3K9me3, HepA occupancy). Our lab showed that under inducing conditions in the wild type (nutrient starvation), H3K9me3 peaks are depleted from the ST BGC, and dissociation of HepA and increase of acetylation levels in the BGC occur, and these events are correlated with transcriptional activation of the cluster [305]. A similar observation was made in *Epichloe festucae* where BGCs that are silent in axenic culture, had H3K9me3 marks, but during plant infection, the H3K9me3 marks were reduced and the BGCs were activated [307]. In contrast, however, the phytopathogenic fungus *F. fujikuroi* does not have any detectable H3K9me3-enriched areas inside BGCs. In *F. fujikuroi*, these marks are generally in pericentric and centromeric regions and sometimes in transposon-rich regions with few or no annotated genes [125]. In *Fusarium mangiferae,* deletion of the *clrD* homolog *FmKMT1* led to increased production of beauvericin biosynthesis while fusapyrone biosynthesis was nearly abolished [308]. These examples suggest that heterochromatin via H3K9 methylation is differently involved in BGC silencing depending on the fungal species and the BGC in question. This pathway may be especially important in fungal species such as aspergilli, which lacks the molecular machinery for the establishment of H3K27me3 (PRC2 complex) associated with facultative heterochromatin in other fungi [309,310,311]. Thus, it is currently unknown if and how facultative heterochromatin is established in these genera that do not possess PRC2 homologs.

#### 5.1.2. Facultative Heterochromatin via H3K27me3 and Polycomb Repressive Complex II (PRC2)

The second pathway for heterochromatin formation is mediated by the polycomb repressive complex II and marked by the histone modification H3K27me3 [307,312,313]. This form of heterochromatin is generally regarded as facultative as it can be conditionally restructured into euchromatin, hence promoting the transcription of underlying genes (e.g., regulation of developmental genes in drosophila) [314]. Some genera such as *Aspergillus and Penicillium* [309], *Ustilago* [310], and some yeasts [311] however are devoid of these chromatin marks. In fungi such as *F. graminearum* [312] and *F. fujikuroi* [315] many BGCs are located in H3K27me3 regions and knockdown or knockout of the methyltransferase-encoding gene *KMT6* of the PRC2 led to de-repression of several BGCs. More interestingly, formerly H3K27me3 regions are acetylated in *KMT6* knock-down strains as shown for the beauvericin BGC in *F. fujikuroi* [315]. Similar effects were observed for *Verticillium dahliae* where lack of Set7, the PRC2 methyl transferase, led to the induction of 839 genes of which 75% are within H3K27me3 domains in the wild type. Furthermore, many genes that are upregulated *in planta* are also located within these domains [316]. In the case of *N. crassa*, H3K27me3 marks are distributed throughout the genome but especially in the sub-telomeric regions where it was shown that a short DNA telomere repeat (i.e., (TTAGGG)_17_) can trigger the establishment of large domains of H3K27me2/3 [317]. In *N. crassa* facultative heterochromatin covers around 7% of the genome containing 1000 silent genes [313]. In *Magnaporthe oryzae*, 50% of TEs and many plant infection-related genes, which are suppressed in axenic culture, are covered by H3K27me3 [318].

These examples show the versatile nature of gene regulation by H3K27me3 and H3K9me3 in different fungal genera which still needs more research that elucidates the mechanism and chronological order of the (de)-repression process.

Accumulating evidence in mammals [319] and also in fungi (e.g., *N. crassa* [317]) suggest that there exist DNA motifs that trigger specific histone modifications. In humans and mouse, over 350 of such motifs have been found and linked to different histone modifications such as H3K4me1, H3K4me2, H3K4me3, H3K27ac, H3K27me3, H3K9me3, H3K9ac, and H3K36me3. Genetic manipulations of these motifs were successful in reducing H3K27 acetylation at a chosen locus [319]. If this is also applicable to filamentous fungi, especially H3K27me3 and H3K9me3 it would be a promising approach for relieving heterochromatic structures around silent BGCs.

Table 6 lists various approaches that lead to the production of SMs by interfering with chromatin active enzymes (via chemicals or via molecular biology methods such as overexpression, deletion, or knockdown).

### 5.2. Euchromatin

While heterochromatic regions suppress the transcription of underlying genes, euchromatin promotes gene expression. It is characterized by highly accessible DNA (i.e., low nucleosome density) and is often marked by histone acetylation and H3K4 trimethylation. Histone acetylation is regarded as an active mark and is established by histone acetyl transferases (HATs) [347]. Usually, they are part of protein complexes such as the Saga/Ada (HAT: GcnE) or the NuA4 complex (HAT: EsaA) [348]. Removal of acetylation marks is performed by histone deacetylases (HDACs) which often mediate transcriptional repression [324]. Some of these HDACs were shown to act on the genome-wide level and are involved in developmental and metabolic processes (e.g., HosA in *A. flavus* [349], HosB and HstA in *A. nidulans* [350]) while others are active in specific regions (i.e., Sir2 in *Candida albicans* in subtelomeric regions [351], HdaA regulates two telomer proximal but not distal clusters in *A. nidulans* [350]). These findings suggest that some HDACs have a location or even cluster-specific activity. Demethylation by HDACs can be inhibited by HDAC inhibitors. Because these chemicals often inactivate multiple HDACs simultaneously, their application results in a genome-wide increase in acetylation and de-regulation of thousands of genes [352]. For references concerning the inhibition or deletion of HDACs for activation of silent BGCs please consult Table 6.

#### 5.2.1. Methylation of Histone 3 Lysin 4

H3K4me1/2/3 is probably one of the most discussed and controversial histone modifications in terms of gene transcription and activation. It is established by the COMPASS complex with Set1 as the catalytic subunit [353]. H3K4me3 is reversed by lysine demethylases such as KdmB in *A. nidulans* [354,355] or Kdm5 in *F. graminearum* [355]. This modification was initially thought to have a transcriptionally promoting character. Recent studies in mice, however, suggest that H3K4me3 is not necessary for transcription but instead prevents silencing of the region by PRC2 or DNA methylation [356]. This would explain several contradictory observations such as the underrepresentation of H3K4me3 in active clusters in several fungal species such as *A. nidulans* [354], *F. fujikuroi* [125] and *F. graminearum* [312] or the partial upregulation of BGCs after abolished H3K4me3 due to deletion of subunits of the COMPASS complex (e.g., *cclA*, *sppA*, *setA*). This was observed in many species such as *A. nidulans* (monodictyphenone, desacetylaustin, dehydroaustinol, citreorosein, emodic acid, 2-OH, 2-aminoemodin, emodin, and the polyketides F9775A and F9775B) [357,358], *A. fumigatus* (gliotoxin) [359], *F. fujikuroi* (BIK, FUS) [360], *F. graminearum* (ZON, fusarins) [360]. But also downregulation of BGCs in *A. nidulans* (Penicillin G, Sterigmatocystin) [355], *F. fujikuroi* (gibberellic acid) [360] and *F. graminearum* (deoxynivalenol) [360] was observed in these mutants. Further puzzling results came from knockout experiments of the H3K4 demethylase (i.e., *kdmB*) in *A. nidulans* by our laboratory. It led to a 20% increase of global H3K4me3 but 90% of all annotated genes that are connected to secondary metabolism were downregulated. Sterigmatocystin and orsellinic acid biosynthesis were reduced while products of the monodictyphenone pathway were increased (i.e., 2,ω-dihydroxyemodin, ω-hydroxyemodin, 2-hydroxyemodin, emodin) [354]. Following this observation, the role of KdmB as inducer of SM clusters goes beyond its function as H3K4m3 demethylase and several indirect effects on general SM regulators may account for the observed phenotype of *kdmB* deletion or overexpression. For example, one could assume that, due to the downregulation of the majority of secondary metabolism genes, the precursor pool may have been increased which may have led to a better turnover by the same amounts of enzymes from the mdp pathway, resulting in higher quantities of mpd-related metabolites [354]. Similar results were found in *F. fujukuroi* [337] and *F. graminearum* [355] but most interestingly, in the case of Kdm5 in *F. graminearum*, we found that many SMs are regulated by Kdm5 in a demethylase-independent role (regulation by a catalytically inactive variant of Kdm5) [355].

Taken together, results that try to link H3K4me3 and active transcription often result in complex and sometimes contradictory results. The role of H3K4me3 as protection against silencing would explain why this mark often but not always is correlated with transcriptional activation. Further insight into the local role of H3K4 trimethylation would be important to evaluate its role in the induction and transcription of silent BGCs.

#### 5.2.2. H4K12 Acetylation

In *A. nidulans*, the H4K12 acetyltransferase EsaA is essential for viability and also involved in the up-regulation of three of four examined BGCs. EsaA overexpression led to depletion of the H4 nucleosome at investigated loci and increased expression levels of BGCs. In the case of the cryptic *ORS* cluster, increased acetylation and nucleosome depletion occurred, but the BGC was not activated. It was suggested by the authors that EsaA and H4K12 acetylation could be responsible for histone depletion at promoter regions so that other effectors such as TFs can access the locus and recruit the transcriptional machinery [361].

Similar findings were published by Roze et al. in *A. parasiticus* where they detected an increase of overall H4 acetylation in the aflatoxin BGC promoters followed by *aflR* binding and transcription [362].

#### 5.2.3. H3K9 Acetylation/H3K14 Acetylation

H3K9 acetylation, which is the mark counteracting silencing by H3K9me3, is enriched in transcriptionally active promoters, sometimes enriched especially in BGCs. In *F. fujikuroi* it was seen that the overall correlation between genome-wide H3K9ac and transcription is weak but that in the case of BGCs the correlation is very strong, suggesting that H3K9ac has important roles in the production of SMs [125]. In the case of the fusarin C BGC, H3K9 acetylation was significantly enriched across the whole BGC when grown under inducing conditions [363]. Another study showed that deletion of the histone deacetylase FfHda1 in *F. fujikuroi* also results in increased levels of H3K9ac in gene promoters and increased transcriptional activity. This even resulted in the activation of the bikaverin cluster under repressive conditions [364].

The most intensively studied HAT GcnE, which is part of the SAGA/Ada complex, was shown to be responsible for the acetylation of H3K9 and H3K14 in *A. nidulans*, and additionally H3K18 and H3K27 in *F. graminearum* [365] and H3K4, H3K9, H3K18 and H3K27 in *F. fujikuroi* [366]. In the case of several BGCs in *A. nidulans* (orsellinic acid, sterigmatocystin, penicillin, terrequinone) GcnE is necessary for activation. While H3K14 acetylation was not enriched within clusters, H3K9 acetylation was only applied in BGCs (only sterigmatocystin and *ORS* clusters were examined for H3K9ac). While genes outside the cluster showed no signs of differential expression, all BGCs that were expressed in the wild type showed abolished transcription in the *gcnE* deletion mutant [367,368]. The acetylation pattern around active genes showed a strong correlation between the increase of H3K9ac around the TSS and transcription while in the case of H3K14ac, the correlation was rather weak. Upon acetylation, the H3 density around the TSS was reduced which suggests that at least one of these marks could be involved in nucleosome depletion around the TSS [367].

Recently the fungal-specific HAT Rtt109, which is responsible for H3K9 and/ or H3K56 acetylation (DNA damage repair), was described in *A. flavus*, *Beauveria bassiana* and *F. graminearum* [369]. Deletion in *A. flavus* led to the removal of both H3K9 and H3K56 acetylation which resulted in impaired growth and virulence, vulnerability to DNA damage stress and also to the abolished expression of genes of the aflatoxin cluster, further promoting H3K9 acetylation as an important mark for gene transcription [370].

#### 5.2.4. H3K27 Acetylation

H3K27 acetylation is the counteracting mark of the silencing H3K27me3 modification. During chromatin accessibility studies in *Neurospora crassa*, it was found that H3K27 acetylation is one of the most predictive histone marks of open chromatin [371]. As already mentioned it was shown in *F. fujikuroi* [335] and *M. oryzae* [318] that H3K27ac spreads to regions previously marked by H3K27me3 if components of the PRC2 complex are deleted. This often resulted in transcriptional activation. Loss of H3K27ac on the other hand did not result in transcriptional inactivation. This suggests that H3K27ac is not necessary for active transcription but plays a role in gene activation/accessibility of previously silenced genes. It seems that H3K27 acetylation by the SAGA complex (i.e., GcnE) is one of the mechanisms that can revert silencing by the PRC2 machinery. The exchange mechanism itself and the involvement of other histone acetylations (by GcnE) in the transcriptional activation is however not fully understood yet.

### 5.3. The Histone Code of Actively Transcribed Genes

Actively transcribed genes have a distinct chromatin signature around the promoter region, gene body, and terminator. We assessed the histone mark occupation around the start codon in all active genes of Chromosome IV of *A. nidulans*. H3K9/K14 acetylation is enriched in the promoter with the highest level at the first nucleosome after the translation start site. H3K4me3 is enriched at the first three nucleosomes downstream of the ATG and H3K36me3 spreads across the active gene body. While H3K4me3 and H3K9/K14Ac decreased towards the 3′ of the gene, H3K36me3 increased [354]. Similar findings were published in *V. dahliae* [316] and *Candida albicans* [351]. Furthermore, it was found that transcriptional active genes correlate positively with increased acetylation within the promoter region in *Aspergillus parasiticus* [362]. Noteworthy, in human cells, artificial application of H3K27 acetylation in the promoter region led to subsequent H3K4me3 and transcription while the deployment of H3K4me3 did not result in increased acetylation or transcription [372].

### 5.4. Finding New Bioactive Compounds by Breaking the Chromatin Silencing Machinery

The production of SMs is a metabolically costly process requiring high energy input to produce the necessary reduction equivalents (NADPH) and ATP. Therefore, their biosynthesis is strictly economized. To ensure that energy is not wasted, fungi by default silence their BGCs at the level of heterochromatin formation, making these genomic regions inaccessible to the transcriptional machinery [305,315,348]. As soon as environmental or developmental conditions demand the production of a certain beneficial SM, the chromatin-based silencing is lifted and specific activation factors start the expression of BGC genes. Experimental setups that aim for induction of fungal BGCs via chromatin modifications either delete, inhibit or overexpress chromatin active enzymes. Most common approaches in this regard are the genetic of chemical inhibition of HDACs [324,331,335,344] or inactivation of components of the silencing machinery via PRC2 [307,312,334,338] or components related to heterochromatin formation via H3K9me (e.g., Dim5/Kmt1/ClrD/HP1, …) [305,307,308,334,339].

One particular successful and easy-to-apply strategy for artificial lifting heterochromatin based silencing was the application of chemical inhibitors for HDACs that are known to be essential for most heterochromatin formation complexes. HDAC inhibitors (HDACi) lead to higher acetylation of histones, a driver for gene expression. The most rewarding results following this strategy used SAHA, butyric acid, valproic acid, nicotinamide, and trichostatin as additives to the growth medium [188,342]. A similar approach that often is applied together with HDACi aims for the inhibition of DNA-methyltransferases (DNMTs). DNA methylation in fungi is mostly connected to the silencing of transposable and repetitive elements [373]. Inhibition or deletion of HDACs or DNMTs resulted in the discovery of nygerone A in *A. niger* [119], or the production of diorcinol 3-O-α-D-ribofuranoside in a marine-derived isolate from *Aspergillus* sp. [130] or in the induction of many silent BGCs during a screen of 162 mangrove endophytic fungi of which 72 produced bioactive substances [137]. Table 6 lists publications that used the genetic or chemical interference of chromatin remodelers for the induction of silent BGCs.

There are however downsides to strategies that interfere with globally active regulators. While approaches that mimic environmental conditions result in a native regulatory response of BGCs, the disruption of global regulatory mechanisms can lead to the synthesis of cryptic compounds that emerge due to uncoordinated regulation of genes or by bio-transformation of the inhibitors [332,344,374]. Additionally, chemical or genetic manipulation of globally active chromatin remodelers can be lethal [315] or lead to phenotypically aberrant transformants (i.e., growth, morphology, conidiation, …) [308,339,375,376,377]. All these factors can result in a substantial change of the whole metabolome [321,346] and transcriptome [326,351] which makes interpretation challenging, even with software solutions that assist with the detection of degradation products or bio-transformed moieties such as BioTransformer [378].

In recent years, however, CRISPR/Cas technology advanced to a point where new options for targeted local chromatin modifications are available and some have already been established in fungal species.

## 6. CRISPR/Cas: New Opportunities in Molecular Biology: Targeted Gene Deletion, Activation and Epigenetic Editing in Filamentous Fungi

CRISPR/Cas is a fast-advancing technology in molecular biology that is nowadays deployed in many prokaryotes and eukaryotes, illustrated by around 25,000 publications connected to “CRISPR” over the last 5 years. Its fast development in terms of genome editing but also in epigenome editing and as a tool for transcriptional activation makes it a very promising option for future application. Although CRISPR/Cas technology is widely used for gene manipulation, approaches for gene and BGC activation are just recently gaining attention in filamentous fungi. In the next section, we shortly introduce the principle of CRISPR/CAS technology and different classes and types that are available before moving to approaches that are applied in filamentous fungi and future perspectives of gene activation (i.e., CRISPRa) applications.

### 6.1. Principle of CRISPR-Cas Technology

The best-known variant of CRIPSR/Cas systems is the RNA-guided nuclease Cas9 from *Streptococcus pyrogenes*, SpCas9. CRISPR/Cas is the major component of the acquired bacterial or archaeal immune system [379]. It is a nuclease that inactivates foreign DNA by causing double-strand breaks at specific DNA-loci via previously acquired guide sequences.

The native lifecycle of the CRISPR/Cas adaptive immunity consists of three stages. Adaption, expression, and interference [380]. Upon initial infection, invading (e.g., phage) DNA is digested by Cas proteins that recognize foreign DNA by a nucleotide sequence that does not occur in the host genome. The so-called protospacer adjacent motif or PAM. Each CRISPR/Cas system has a distinct PAM sequence. A short stretch of the DNA (i.e., protospacer) is then cleaved out and inserted into a DNA locus called CRISPR-array. In the expression phase, the whole CRISPR array with all protospacers is transcribed (i.e., pre-CRISPR RNA or pre-crRNA) and processed into mature CRISPR RNAs (i.e., crRNA). Different CRISPR/Cas variants have different procedures during this phase, either demanding a complex interplay of many Cas proteins or requiring only one multi-domain protein [380]. During the interference stage, the mature crRNA or guide RNA is used as a reference pattern for the Cas nucleases to inactivate invading non-host DNAs by inducing double-strand breaks at loci that match the crRNA sequence and possess a PAM motif [380].

After the discovery of CRISPR/Cas, its potential instantly attracted the interest of the scientific community. Many research groups refined this strategy over the past years to one of the most versatile tools used in modern molecular biology that has now been established in many eukaryotic organisms, including filamentous fungi [381,382]. New variants of the CRISPR/Cas from different bacterial sources or engineered forms evolve(d). Some are only capable of nicking the DNA or even are incapable of modifying the DNA at all (nuclease- dead Cas9, dCas9). By fusing these variants with proteins of versatile functionality, researchers created a multitude of effector proteins that can be targeted precisely throughout the genome. This opened many new possibilities for addressing research questions that go beyond genome editing, such as CRISPR/Cas mediated chromatin modification [252], gene activation (CRISPRa) [251,253,271,383] or transcriptional interference (CRISPRi) [384,385] and locus-specific protein proximity labelling (CasID or BioID) [386].

### 6.2. Differences between CRISPR/Cas Variants

Following Cas9, different variants of CRISPR-Cas systems have been found in nature as reviewed in detail by [380]. They are divided into two classes which differ in their complexity. While class I CRISPR/Cas systems utilize many proteins for each phase of the natural defense (adaption, expression, interference), class II CRISPR/Cas systems only use 2–3 proteins during adaption and only 1 (Type V (i.e., Cas12a) and VI) or two (Type II (i.e., Cas9)) proteins during expression and interference. The simplicity of Class II systems makes them the prevalent choice for CRISPR/Cas applications in molecular biology. Depending on the type of Cas variant, they can either target RNA molecules (Type VI) or DNA (Type II and V) molecules [381]. Within Class II especially SpCas9 from *Streptococcus pyrogenes* or Cas12a (i.e., Cpf1) from *Francisella novicida, Acidaminococcus* sp. or *Lachnospiraceae bacterium* (i.e., FnCas12a, AsCas12a, LbCas12a) are being deployed for molecular biology in fungi. They have important differences that make both of them viable options, depending on the application [381].

**Figure 3:**
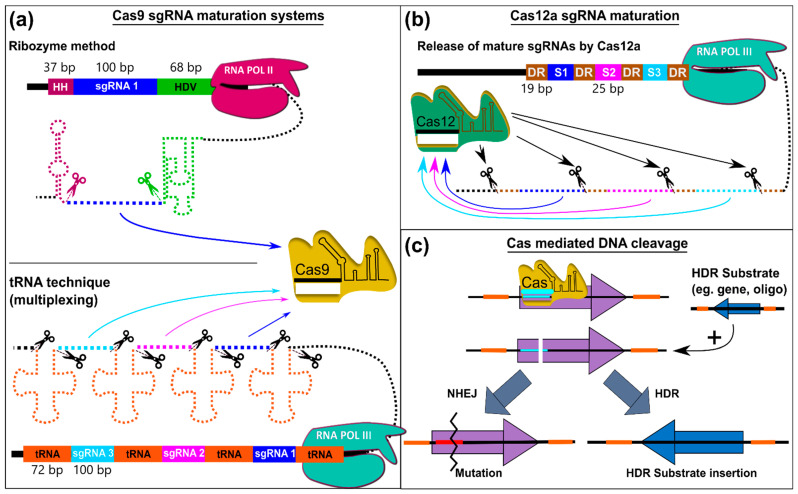
Maturation of guide RNAs and Cas mediated Gene editing. (**a**) Methods for release of mature sgRNAs with Cas9. **Top**: the ~100 bp long sgRNA is flanked by the 37 bp Hammerhead (HH) and the 68 bp HDV ribozyme. Upon transcription by RNA polymerase II (RNA POL II), the sgRNA is released by the formation of secondary structure and autocleavage of the ribozymes. **Bottom**: Cas9 sgRNAs can be matured and released by flanking it by ~72 bp long tRNAs (i.e., glycine). Upon transcription by RNA polymerase III (RNA POL III), the tRNAs are cleaved by the endogenous RNAse P and RNAse Z and the mature sgRNA is released which then can form a functional complex with Cas9. Arrays of sgRNA and tRNAs can be cloned and expressed in vivo for multiplexing purposes. (**b**) Cas12a can process the CRISPR transcript itself. The expression fragment contains an only ~19 bp direct repeat followed by the approximately 25 bp long protospacer sequence. Due to its small size, it is easy to clone several guide RNAs in sequence for multiplexing purposes. After expression by RNA polymerase III (RNA POL III), the transcript is processed by Cas12a and the mature sgRNAs are released which can form a functional complex with Cas12a. (**c**) two options for gene editing with CRISPR/Cas technology. After the successful introduction of the double-strand break, the DNA can either be repaired via the non-homologous end joining pathway (NHEJ) or, if an oligo or gene with complementary regions (orange) is provided (i.e., HDR Substrate), the homology directed repair mechanism can be activated (HDR) and the provided genetic material is introduced into the target locus.

Easier handling and cloning with Cas12a due to different gRNA processing. Cas9 needs the matured form of the tracrRNA and the crRNA which, in nature, anneal to produce a functional guide RNA that can then build a complex with the Cas9 protein. Genetic engineering-led, however, to the development of both crRNA and tracrRNA in one single guide RNA (sgRNA) which increased the handling comfort and efficiency [387]. Nevertheless, for the proper maturation of the sgRNA, it has to be ensured that only the sgRNA without any additional nucleotides is released from the transcript which is currently accomplished by either ribozymes [388] or tRNAs [389] that flank the sgRNA. The sgRNA maturation via ribozymes uses an RNA polymerase II promoter and terminator together with the Hammerhead and HDV ribozymes to release the mature sgRNA [388]. Nødvig and co-workers took this idea even further and developed a system capable of multiplexing sgRNAs utilizing the endogenous tRNA processing machinery. It consists of a cassette that uses an RNA polymerase III promoter to express sgRNAs which are flanked by tRNAs. This then results in the release of mature sgRNAs by tRNA-specific RNAse P and RNAse Z enzymes [389] (Figure 3a). This made it possible to express multiple sgRNAs at the same time thereby increasing editing efficiency by application of several gRNAs at the same locus which in turn allows for multiple gene editing. However, the genetic scaffold that is required for proper processing and release of multiple mature sgRNAs makes the cloning and transformation process of Cas9 multiplexing systems a challenging and labor-intensive endeavour in filamentous fungi. In the case of the ribozyme approach, the repetitive expression scaffold for one gRNA sequence is around 185 nt-long [388] and in the case of the tRNA-assisted approach, it spans even around 222 nts (containing 2x identical glycine tRNA) [389] (Figure 3a).

In stark contrast to Cas9, Cas12a can directly process the U6 promoter-driven CRISPR array into the mature guide RNAs which only consists of a 19 nt-long direct repeat (DR) and a 23–25 nt-long protospacer sequence (PS) (i.e., U6-DR-PS-DR-PS-DR-PS-, etc.) (Figure 3b). This greatly facilitates the cloning and application of single and multiple sgRNAs with Cas12a [390,391,392,393].

The DNA cleavage pattern is different between Cas9 and Cas12a. While SpCas9 produces a blunt end cut 3 bps upstream of the NGG PAM [387], Cas12a variant FnCas12a cleaves after the 23rd base on the targeted strand at the 18th base of the non-targeted strand which results in staggering cleavage sites distant from the PAM [394]. In the case of Cas9, the cleavage is mediated by two nuclease domains (HNH and RuvC) [395], while Cas12a only has one RuvC domain for the cleavage of both strands [394].

The PAM sequence of Cas9 is the short 3 nucleotide long G-rich motif NGG while Cas12a has a T-rich PAM (AsCas12a and LbCas12a: 5′-TTTN-3′ [381] or 5′-TTTV-3′ [396]; FnCas12a: 5′-TTN-3′ [394]. Other PAMs are possible, depending on the origin of Cas12a [397]. There are also engineered variants that accept a broader range of PAMs which will be described in Section 6.5. The PAM greatly limits the loci that can be targeted because, without a present PAM, neither annealing nor cutting occurs properly.

Genetic engineering led to the development of catalytically inactive (i.e., dead or dCas) and nicking CRISPR/Cas tools. Cas9 proteins that can only nick the DNA (i.e., nCas9) [398] resulted in improved single base editing efficacy [399]. There are also mutant versions of Cas12a proteins although they are not yet capable of perfect nicks as they still retain some of the catalytic activity for the second strand. Merely the ratio of single and double-strand breaks is shifted in favor of the nicks [400,401]. In contrast to Cas9, Cas12a only has one catalytic domain that cleaves both strands which makes the engineering of nCas12a variants more complicated.

The development of catalytically inactive Cas9 and later Cas12a variants (i.e., dead or dCas9 and dCas12a) made CRISPR/Cas to a very powerful tool in molecular biology [400,402]. dCas proteins can be used as guidance vessels for attached proteins or protein domains. This way, CRISPR/Cas can be deployed for a multitude of applications, involving basically any protein that can be functionally fused to the N and/or C-terminal or even as a mediator of transcriptional interference (CRISPRi) by the sterical hindrance of the transcriptional machinery within a gene body or at the transcriptional start site [403].

Although SpCas9 is currently the prevalent choice for gene editing and other applications, the Cas12a system has many benefits for working with filamentous fungi. The biggest advantage with regard to BGC activation is the easy deployment of multiple gRNAs as compared to SpCas9. While Cas12a can process the raw pre-crRNA transcript directly, Cas9 needs more complex cloning strategies involving features that allow for a proper release of blunt end sgRNAs (e.g., ribozymes or tRNA scaffolds). Cas12a is, therefore, much easier to handle which not only makes experiments more time efficient but also more robust as the system requires fewer components.

The second advantage for work with filamentous fungi specifically is the temperature maximum. Most filamentous fungi grow well around room temperature. SpCas9 has its catalytic maximum at 32 °C which can be challenging for some fungi. Cas12a, on the other hand, can be deployed at lower temperatures (i.e., 28 °C) which increases the efficiency in most fungal strains [404].

One of the drawbacks of the CRISPR/Cas system is the requirement of the PAM motif at the target locus. The difference in PAM sequences in different variants increases the versatility and flexibility in this matter which can be very important for experiments that require specific loci, with limited PAM options, to be targeted.

### 6.3. Gene Editing via CRISPR/Cas

Various approaches that involve CRISPR/Cas technology have been already applied to different fungal species [395]. Especially in fungal species where site-directed mutagenesis and gene editing rates by conventional methods are very low, CRISPR/Cas is a promising option [405,406].

To increase the editing efficiency by utilizing homologous DNA repair pathways, linear or circular double-stranded DNA with homologous regions or single-stranded DNA oligos with small homologous regions can be transformed together with the Cas construct. These then can get inserted at the Cas9 restriction site after cleavage which not only can increase the mutation rate but makes it also possible not only to mutate, but exchange the DNA sequence via homology-directed repair mechanisms (Figure 3c) [389,407].

The transformation of the CRISPR/Cas machinery and the guide RNA(s) can be performed on a single [388] or multiple plasmids [251], episomal plasmids by utilization of the AMA1 region of *A. nidulans* [388,389], by random integration into the genome [408] or within a specific genomic locus [251]. We recently also developed an inducible CRISPR/Cas variant in filamentous fungi that uses the human estrogen receptor [251,409]. Other variants utilize the Tet-ON system for induction [410].

If the fungus is not amenable to stable or transient genomic transformations, mutation by CRISPR/Cas systems can also be achieved by transformation of the in vitro pre-assembled (i.e., sgRNA and Cas-protein) ribonucleotide complex (RNP) [407,411,412]. For more information about CRISPR/CAS applications in fungi, please consult the recent review by Schuster and Kahmann [413].

### 6.4. Gene and BGC Activation via CRISPR/dCAS Variants

**Figure 4:**
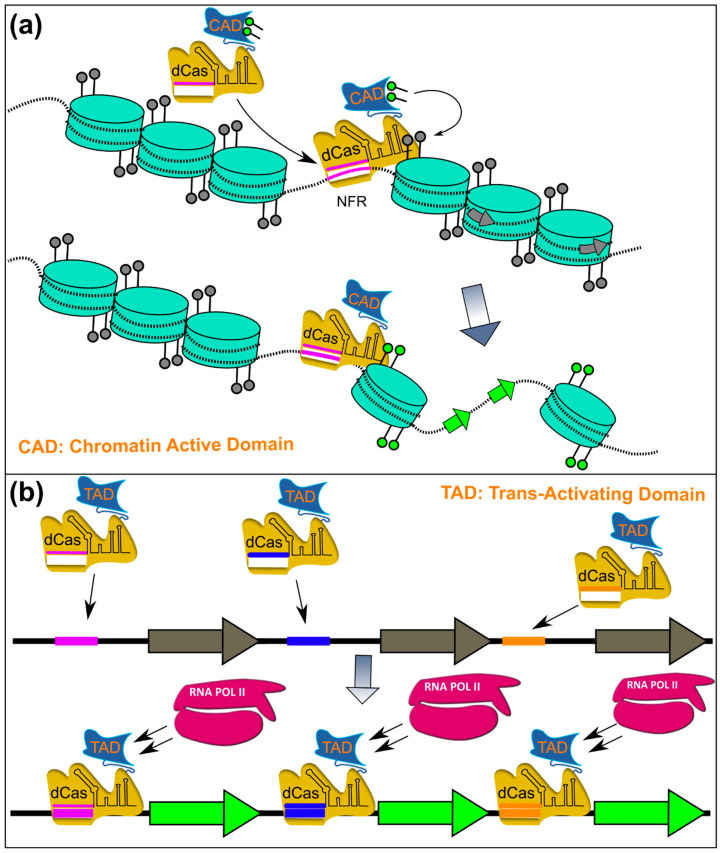
Targeted gene activation approaches via CRISPR/Cas technology. (**a**) targeted chromatin remodeling via a chromatin active domain (CAD). DNA is depicted in dotted black lines, nucleosomes are cyan, heterochromatic histone modifications are grey pins while euchromatic histone modifications are green pins. A fusion protein of dCas and a CAD is targeted into a nucleosome-free region (NFR) in a heterochromatic area where it modifies the local histones. This leads to an increase in DNA accessibility and promotes transcription. (**b**) Targeted gene activation by dCas fused to a trans-activating domain (TAD; e.g., VPR). DNA is depicted as a black bar, genes are either grey (inactive) or green (active) arrows. The colored bars in front of the genes are target sites for dCas-TAD (i.e., protospacer sequence). dCas-TAD is sent into accessible promoter regions of target genes. The TAD recruits components of the transcriptional machinery which leads to the transcription of the target gene. By using multiple guide RNAs, several genes/promoters can be targeted simultaneously.

Catalytically inactive Cas variants (i.e., dCas9, dCas12a) have great potential to be used for the activation of silent BGCs. When fused to a trans-activator domain it can be deployed as a programmable TF for the activation of one or multiple genes (via sgRNA multiplexing; Figure 3a,b) [251,253]. Another potent approach is the fusion of a chromatin remodeler (domain) which would provide the possibility to change histone modifications in a targeted and locus-specific manner [252]. This way, BGCs that are buried within heterochromatin could be made accessible by replacing the silencing marks (i.e., H3K9me3, H3K27me3) with active marks (i.e., acetylation). Figure 4 illustrates both dCas-mediated approaches for the activation of silent BGCs.

#### 6.4.1. Transcriptional Regulation

The fusion of a trans-activator or trans-activating domain and a dCas protein transforms it into a programmable transcription factor that can be sent to any spot within the genome (as long as an appropriate PAM is present). This CRISPRa method was established simultaneously in the filamentous fungi *Aspergillus nidulans* for dCas9 [251] and dCas12a [253] and shortly after also in *P. rubens* [271]. In every case, the trans-activator VPR [414] was chosen as it was shown to be more effective than other known trans-activator domains. It is a tripartite activator with protein domains that were shown to interact with parts of the transcriptional machinery and also the histone acetylase complex SAGA [415]. The hybrid trans-activator consists of four copies of the VP16 domain of the Herpes simplex virus, the p65 domain of the human activator NF-κB and the Rta domain of the Epstein-Barr virus TF. These publications tackle multiple challenges when it comes to BGC activation. Roux et al. successfully deployed dCas12a-VPR in an episomal and stable approach. They showed that the activation of a BGC can be achieved by upregulation of the pathway-specific TF but also by using guide RNA multiplexing to upregulate all genes of a BGC [253]. Simultaneously, we published a nucleosome positioning map of the genome of *A. nidulans* which was used to target VPR-dCas9 into nucleosome-free regions within the promoter [251]. This facilitates the binding of VPR-dCas9 as it was shown that steric hindrances such as nucleosomes or other proteins can prevent the annealing of (d)Cas proteins to their target [416]. Furthermore, we deployed an inducible version of VPR-dCas9 to precisely time the activation process because some metabolites could be toxic to the host and therefore interfere with the morphology and (chemical) phenotype of the fungus if the BGC product is synthesized constitutively [251].

#### 6.4.2. Targeted Chromatin Remodelling Using CRISPR/Cas

Targeted locus-specific deposition of histone marks via fusion proteins of dCas and chromatin active domains is an emerging field in molecular biology. In human cancer cells, targeted modification of H3K9me3 and H3K27ac can be accomplished by deploying a fusion protein composed of dCas9 and the H3K9 methyl-transferase KRAB (i.e., Krüppel-associated box repressor) or the histone acetyl transferase p300, respectively. In both cases, the targeting of the fusion proteins to DNA accessible sites (e.g., nucleosome-free regions, NFRs) led to the respective modification at the locus (i.e., H3K9me3 and H3K27ac). In the case of H3K9me3, it was even shown that the DNA is inaccessible after the histone mark H3K9me3 is deployed [417,418].

In fungi, gene activation and repression by targeting chromatin active domains via CRISPR/Cas to target loci were accomplished in *A. niger,* although the actual effect on chromatin status (i.e., histone modifications) has not been assessed [252]. Li et al. hereby targeted the human acetyl transferase p300 to the promoter region of the HR-PKS-encoding gene *FUM1* and managed to activate the fumonisin cluster in *A. niger*. Furthermore, they fused endogenous proteins such as GcnE, the acetyltransferase of the SAGA complex, histone deacetylases HosA and RpdA, and the H3K9 demethylase Lde to dCas9 separately and tested its effect on the SM gene *breF*. While the fusion of the catalytic core of human p300 fused to dCas9 led to an activation of the target gene as expected, fusion proteins of dCas9 and GcnE, RpdA and HosA resulted in repression of the gene while no difference was seen with the dCas9-Lde fusion protein. While most of the results are as expected, the repression effect by dCas9-GcnE is interesting. Although the authors state that the repression of *breF* by dCas9-GcnE is expected as GcnE was shown to be a negative regulator of 12 PKSs in *A. niger*, the result is surprising to us. Upregulation of BGCs due to the deletion of a broad domain regulator-encoding gene such as *gcnE* could be due to many indirect effects, for instance if GcnE was the activator of a repressor of these BGCs. Targeting the acetyltransferase to a specific genomic location, however, should lead to higher levels of acetylation only at the specific location and, therefore, to upregulation of only the targeted gene.

In the case of the dCas9-HosA, activation of the pigmentation gene *fwnA* was observed. Again, this is theoretically consistent with literature where *fwnA* expression decreased in *hosA* deletion strains. Such as with GcnE however, comparing a local effect (i.e., deacetylation of the promoter region) with a genome-wide effect (i.e., deletion of broad domain regulator such as HosA) does not take indirect regulatory effects into account. Unfortunately, histone modifications of the targeted loci were not analyzed, so it is not clear if really a change in histone modifications is responsible for the observations. Furthermore, there could also be the possibility of host-mediated recruitment of the endogenous fusion protein (i.e., HosA, RpdA, GncE, Lde) which could interfere with the experimental setup. Using only the catalytic domain (of homologs) would be a way of circumventing this.

Histone phosphorylation is another mechanism for gene induction that is prevalent in stress response gene promoters. A fusion of dCas9 and a hyperactive variant of the human histone kinase MSK1 (i.e., dMSK1) could be targeted to promoters for gene activation by phosphorylation of H3S28 and H3S10 [419]. Here, several (also non-stress response) genes were activated by targeting the promoter. When gene activation was successful, an increase in H3S10ph, H3S28ph, and H4K27ac was found. In cases where H3S10ph but not H3S28ph occurred, neither H4K27ac nor gene activation was observed. This was, however, not yet tested in fungal organisms.

In summary, the targeted deployment of histone modifications as a regulator of transcription is an emerging field in fungal research but experimental data is currently scarce. More research is needed to fully assess this strategy for the activation of silent BGCs. If successful, CRISPR/Cas-based targeted deployment of chromatin active domains will not only result in the discovery of many natural products but will also provide a lot of information about transcription and the local chromatin context.

### 6.5. Application Relevant Information for CRISPR/Cas-Methods

Next, we want to summarize insights regarding the deployment of CRISPR/Cas methods. This is aimed at helping with the experimental planning. It will focus on the two main CAS systems that have been used for fungal research, Cas9, and Cas12a.

Cas and dCas approaches have different prerequisites in regard to the target site, residence time and off-targets. When using CRISPR/Cas for gene-editing purposes, the successful outcome depends on a one-time event (the cleavage of DNA). In the case of CRISRRa or CRISPRi however the effect is determined by a continuous interaction of the fusion complex with the proteome or DNA at the target locus [420,421]. Concluding, a sgRNA that proved to elicit DNA mutations via CRISPR/Cas might not be of use for experiments where a long residence time is essential (e.g., CRISPRa).

Off-target annealing also has different consequences depending on the application. Cas proteins can anneal to off-target sites but as long as the sgRNA does not match appropriately, no cut will occur. In the case of dCas approaches, however, this could theoretically lead to off-target effects mediated by the active domain that is fused to the dCas protein.

There are several tools that design gRNAs for different (d)Cas systems based on one or several of the following features: sequence composition, chromatin accessibility, gene expression, nucleotide position, RNA secondary structure, melting temperature, free energy, off-target possibility, and more [422]. Some of them also feature different application types such as DNA cleavage, CRISPRi and CRISPRa. This is important because the sgRNA/crRNA design for mutational Cas approaches is different from those for CRISPRa approaches because they target the gene body and not promoter regions [423]. Most of them are biased towards one or more organisms (mostly mammalian) or applications (e.g., mutational vs. CRISPRa). Some tools such as Cas-Designer (Cas9) [424] feature a broad spectrum of genomes that are continuously updated (also by user request). CASPER (Cas9 and Cas12a) is not biased towards an organism and was specifically designed for genome editing in non-model organisms [425]. Guide RNA design tools for non-model organism often calculate the gRNA efficiency based only on sequence features which can lead to reduced sgRNA performance if other, unconsidered parameters, such as nucleosome occupancy, prevents target site accessibility.

In the case of CRISPRa approaches, the location of deployment within the promoter (in plants) was shown to have a distinctly different effect on the activation of the target gene even if the sgRNAs were only as little as 50 base-pairs apart from each other [426]. For more detailed information, read the following recent reviews which describe available prediction tools and guide RNA optimization [422,427].

Cas efficiency depends on multiple factors including Cas-sgRNA complex formation and concentration, target site accessibility, residence time, and codon optimization. By codon optimization alone, Cas efficiency could be increased from 35 to 70% [428].

Cas stabilizes the sgRNA, but the complex formation can be hindered by other RNAs and some divalent ions. To be functional, the Cas protein and the sgRNA/crRNA have to form a complex. Unbound sgRNAs get degraded very quickly. Upon complex formation, the Cas9 protein stabilizes the sgRNA, undergoes conformational changes, and is released from the inactive apo-state [429,430]. Random RNA molecules that are present in the cell also can bind unspecific to Cas9 and compete with sgRNAs which delays or prevents complex formation [431]. After complex formation however, unspecific RNAs have no significant impact on the ribonucleotide stability anymore [429]. It is therefore important to express the sgRNA/crRNA at high levels so that the Cas protein has a high chance of collision before another RNA molecule can interfere or the sgRNA/crRNA is degraded [429]. The following publications give in-depth information about Cas9 [432] and Cas12a [433] kinetics.

It also is important to note that in the apo-state Cas9 can bind DNA in a non-specific manner while the protein remains catalytically inactive, so no unguided “rogue”-activity is happening under normal conditions [430]. It was, however, shown that the presence of Mn^2+^ or Co^2+^ ions can lead to unspecific DNA cleavage even without bound crRNA (Cas12a) or sgRNA (Cas9), respectively, which makes media composition potentially also an important brick in the wall of CRISPR/Cas applications [434]. Processing of the pre-crRNA by Cas12a variants does not seem to be Mg^2+^-dependent but it was shown that in the case of LbCas12a and FnCas12a, affinity to the crRNA is dependent on Mg^2+^ ions which were found to be coordinated in the RNP complex [435,436]. It can be concluded that divalent ions can have a significant impact on Cas activity and specificity which should be kept in mind when deciding on culture conditions.

The determinants of the residence time of Cas at its target locus are the proper pairing with the PAM, the subsequent annealing to the seed region, the local proteome and its fluctuations (i.e., accessibility and dislocation), and the concentration of Cas-sgRNA complex [421]. The impact of mutations on the annealing, residence time and cleavage efficiency also follow this order (PAM > seed sequence > PAM-distal sgRNA sequence (=5′-end)) with the proteomic occupancy around the target locus having a varying influence [429,437]. The residence time is significantly shorter the less complementary the annealing sequence is which was shown in human cell lines where residence times of less than two minutes to up to more than three hours have been observed [429].

The proteome of the target site can prevent binding of Cas-sgRNA complex or even dislodge bound Cas. In vitro experiments confirmed that cleavage by Cas9 is nearly abolished when targeted to DNA occupied by nucleosomes. At the edges of nucleosomes or within linker regions, however, cleavage was largely unaffected. Interestingly, the position of the PAM was the essential determinant of cleavage efficiency when sequences close to the border of a nucleosome were targeted. This was verified in vitro by designing two nearly perfectly overlapping sgRNAs (shifted by four nts) but with opposite orientations. The sgRNA that had the PAM located proximal to the nucleosome showed drastically reduced cleavage efficiency compared to the sgRNA that had the PAM located distal [416]. This observation was also confirmed in vivo in human cell lines [438,439,440]. The magnitude of this effect is, however, also dependent on the strength of the interaction between nucleosome and DNA. Upon comparing two different DNA sequences that are known to have different affinities to nucleosomes, it was shown that breathing chromatin structures (fluctuations of DNA occupancy of the nucleosome) show a much higher cleaving permissiveness towards Cas9 than nucleosomes with strong interactions to DNA. Interestingly, the cleaving efficiency around the nucleosome dyad was comparably low in both cases. Furthermore, when repeating the same experiments with the addition of the chromatin remodeler SNF2H or RSC, Cas9 cleavage efficiency was significantly enhanced [441].

The obstruction of CRISPR/Cas by proteins is not limited to nucleosomes. In the case of SpCas9 (*Streptococcus pyogenes*), which is known to remain bound to both DNA ends after cleavage [442], RNA polymerase was able to dislodge the Cas9 protein when targeted to the template strand. This resulted in increased editing efficiency due to the release of both cleaved DNA ends and the onset of the DNA repair machinery [443]. This might however only be true for SpCas9 because in the case of SaCas9 (*Staphylococcus aureus*) [444] and AsCas12a (*Acidaminococcus* sp.) [445] it was shown that these proteins automatically release one DNA strand some hours after the catalytic event.

Apart from the sgRNA annealing site itself, post-PAM interactions were found to be important for stable interactions between the Cas protein and the DNA. SpCas9 was not able to remain in a place of the target site when the DNA sequence 14 base pairs downstream was occupied by another protein. It was observed that this post-PAM interaction is weak in force but very crucial for Cas9 binding and cleavage [446]. Displacement mediated by disturbance of the post-PAM accessibility can also happen after the association of SpCas9 to the DNA. In the case of SaCas9, the post-PAM interaction was only around seven base-pairs and had a much lesser effect than on SpCas9 [446]. SaCas9, in stark contrast to SpCas9, represented a significant barrier for other proteins such as DNA-tracking motors [444]. Zhang and co-workers give a nice comparison of interactions strength between SaCas9 and SpCas9 mediated by different regions across the footprint [444].

Methylated DNA could be cleaved without problems by Cas9 [447] and also CRISPRa approaches had been successful when the target area had DNA-methylated sites [442].

The examples from above show that the proteome of the target site can have a profound impact on the binding and residence time and therefore cleavage efficiency of CRISPR/Cas. Structures that statically occupy and block the DNA such as nucleosomes should be avoided when choosing a target site for CRISPR/Cas applications.

Off-target annealing has different consequences, depending on the application (i.e., Cas vs. dead-Cas). Cleavage at off-target locations depends on PAM sequence and guide-RNA homology. Two major concerns about the deployment of CRISPR/Cas systems are the possibility of off-target effects (i.e., annealing to a not intended stretch of DNA) and the restriction that CRISPR/Cas systems need a specific PAM sequence for annealing. An interesting finding in case of off-target activity of Cas9 was, that the RNP complex could bind to many off-targets, even with a seed sequence complementarity of only five nts (seed: 6–12 nts or 5 nts proximal to the PAM sequence for SpCas9 [448] or FnCas12a [394,435] respectively). However, for proper catalytic function, a complementary gRNA of at least 17 nts was found to be necessary. In the case of Cas9, proper initial binding of the Cas9-sgRNA complex to the DNA is mainly mediated by PAM interaction and complementary forces between the DNA and the seed region of the sgRNA. In a second step, Cas9 cleaves after sufficient pairing of the 5′-end of the guide RNA [449]. This model would explain why binding of Cas9 to the target site can even occur in case of little homology between sgRNA and target site but cleavage is nevertheless impaired due to low overall homology between sgRNA and target sequence.

The PAMS and, therefore, the possible annealing/cutting sites are different for Cas9 and Cas12a and are one of the biggest limitations of these tools. As already mentioned, Cas9 has a G-rich while Cas12a variants have T-rich PAMs. To circumvent these limitations, protein engineering performed on Cas9 and Cas12a led to the development of near- PAMless [450] or PAM-flexible [451,452,453,454] variants with increased off-target activity. But also high fidelity (HF) [455] variants have been engineered. Even combinations of both variants exist which compensate for the increased off-target activity of near-PAM-less or PAM-flexible variants [453,454]. It is of importance that the sgRNA/crRNA design has to account for the flexible PAM. It must not enable the Cas protein to anneal to the gRNA expression cassette itself which could lead to cleavage or transcriptional blockage. To assess the (off-target) cleavage pattern of a specific Cas-sgRNA complex *in vivo*, the GUIDE-SEQ approach can be applied which performs an unbiased detection of CRISPR/Cas-mediated double-strand breaks (DSBs) by in vivo integration of an oligo at sites that have DSBs (i.e., on- and off-target loci) and subsequent amplification and next-generation sequencing [456].

The information above should illustrate the many application forms and variants of CRISPR/Cas systems. There is not a one for all experimental strategy because the prerequisites of the experimental question determine not only the variant of CRISPR/Cas to be deployed but also the target genetic features (i.e., gene body in case of gene knockouts but promoters in case of activation via CRISPRa approaches) and their connected properties (i.e., PAM, proteomic occupancy, sequence bias) that have to be considered during gRNA design. In fungal organisms, mutational CRISPR approaches are already widely implemented. In the case of CRISPRa or targeted chromatin remodeling, however, this technology is still in its infancy but harbors great potential of the activation of BGCs and the discovery of new bioactive metabolites in the future.

## 7. Perspectives

Genome mining is a core component in the revelation of unknown BGCs in fungal genomes. The data sources that are used for predictions are very diverse across the platforms and include not only DNA or protein sequences but also transcript data, phylogenetic data, and more. Deploying more and more predictive parameters that are connected to BGCs and their regulation will surely promote the discovery of new BGCs in the future. Increasing insights into novel BGC types (i.e., gene types, protein domains, genomic arrangement, backbone genes, etc.) will also lead to a transition from “high novelty/low reliability” to “low novelty/high reliability” BGC prediction algorithms which, in turn, again increases their discovery rate. Approaches for the detection of novel BGC types are very diverse and will surely enable us to discover new and currently unknown BGC types in heavily researched model organisms.

The development of these tools, however, is similar to a feedback loop between prediction and experimental verification. Predictions of known BGCs largely depend on the detection of patterns from already verified BGC types and so, the provision of high-quality experimental verification data of BGCs is a crucial component of reliable predictions. Curated databases fulfill these requirements but need the regular attention of professionals in the field. The guided deposition of such data by the research group itself into curated databases will hopefully become a standard procedure within the publication process in the future. This would increase the reliability of predictions and also accelerate the discovery process of BGCs in fungal genomes and, hence, the development of novel pharmaceutics.

CRISPR/Cas technology is emerging as a new tool and already has proven to deliver new molecules by the activation of silent BGCs in a number of organisms. Its functionality as a transcriptional activator was already experimentally shown in fungi by fusing the trans-activator VPR to dCas9 and dCas12a. Heterochromatic regions, however, could get problematic because of target site accessibility and the sole deployment of trans-activator domains.

The targeted local histone modifications could represent the next step in the toolbox for the activation of silent BGCs. If it is possible to relieve heterochromatic regions of silent marks (i.e., H3K27me3, H3K9me3), the targeted and locus-specific transition to transcriptionally active euchromatin could be within reach. The choice of the right histone-modification domain, however, is still a topic that needs thorough research. Many observations that connect transcriptional activation and histone modification indicate that H3K9 acetylation (gene activation especially in the case of BGCs), deployed by Rtt109 and GcnE, and H3K27 acetylation (hallmark for open chromatin and de-repression in *N. crassa*), also deployed by GcnE, within the promoter region correlate best with gene activation across several fungal species. As there are often nucleosome-free regions in promoters, even within heterochromatin, nucleosome positioning maps assist with the choice of a proper entry point of dCas-fusion proteins. Depending on the target locus and the prevalent histone marks, additional activators (e.g., VPR) could be necessary to induce target genes. H3S28ph also seems a promising way of activating single genes but more research, especially in the fungal context is needed in this regard. Furthermore, if DNA motifs are also proven to be responsible for occurring histone marks in fungi, this would be an additional opportunity to relieve whole BGCs from heterochromatin. This way, the programmable histone modifier can be used in combination with these mutants to activate silent BGCs and reveal their biosynthetic product.

## Figures and Tables

**Table 1:** List of platforms and databases used for BGC prediction. Ordered by the most recent update. Status March 2022.

Tools and Database Used for BGC Predictions					
Tool/ Database	Type	Purposes	Last Update	Link (Accessed on 15 March 2022)	Ref.
EMBL-EBI/ENA	DB	uncurated Database (large repository: DNA, RNA, genome annotations)	regular updates	ebi.ac.uk	[87,88]
GenBank	DB	uncurated Database (large repository: DNA, RNA, genome annotations)	every 2 month	ncbi.nlm.nih.gov/genbank	[89]
GNPS	DB	“Global Natural Products Social Molecular Networking” community-curated mass spectrometry database	2022	gnps.ucsd.edu	[90]
fungiSMASH	PT	Gold standard for BGC prediction. Allrounder with an extensive variety of integrated tools and algorithms, web-application or stand-alone; Rule-based and rule-independent algorithms. Versions for fungi, bacteria and plants available. Fungal version has additional BGC border detection based on TF-binding sites	2021	https://fungismash.secondarymetabolites.org/#!/start	[78]
NP-atlas 2.0	DB	curated community database of microbially-derived SMs compounds from marine macro algae and diatoms are excluded	2021	npatlas.org/	[91]
TIGRFAMs	DB	curated database of protein families (mainly prokaryotic)	2021	ncbi.nlm.nih.gov/genome/annotation_prok/tigrfams/	[92]
PFAM v35	DB	partly curated database protein domains and families	2021	pfam.xfam.org/	[93]
FunOrder	PT	detection of essential genes within a BGC by a co-evolutionary approach	2021	github.com/gvignolle/FunOrder	[72]
ARTS 2.0	PT	Antibiotics cluster prediction by detection of resistance genes; web application	2020	arts.ziemertlab.com	[83]
HMMER	PT	DNA based Homology search via pHMM; as source code or as web application	2020	hmmer.org/	[94]
CO-OCCUR	PT	genome-wide co-occurence of BGC-related gene pairs; source code available	2020	github.com/egluckthaler/co-occur	[37]
TOUCAN	PT	BGC prediction via phylogeny, function, composition at amino acid level (k-mers, Pfam protein domains, and GO terms); source code (python/perl) available	2020	github.com/bioinfoUQAM/TOUCAN	[95]
antiSMASH Database v3	DB	high-quality genomes and BGCs of bacteria, archaea, and fungi	2020	antismash-db.secondarymetabolites.org/	[96]
NORINE	DB	manually curated DB for NRPS	2020	bioinfo.lifl.fr/norine/	[97]
FRIGG	PT	Antibiotics BGC prediction based on presence of resistance gene; Pipeline that combines different tools; Python and R-based script available	2019	zenodo.org/record/2560245#.YjlMHepBw5s	[85]
BIG-SCAPE + CORASON	Output refinement	takes output of antismash and generates phylogenic BGC family networks; software application	2019	git.wageningenur.nl/medema-group/BiG-SCAPE	[98]
MiBIg 2	DB	curated database (community effort and institute curation) of known BGCs and their products	2019	mibig.secondarymetabolites.org/	[99]
IMG-ABC v5.0	DB	predicted BGCs with environmental metadata (prediction via antiSMASH v5)	2019	img.jgi.doe.gov/abc-public	[100]
Deep-BGC	PT	Deep learning strategy to determine BGC product classes and chemical activity	2019	github.com/Merck/deepbgc	[101]
RiPPMiner	PT	RiPPs; web-application	2017	nii.ac.in/rippminer.html	[102]
SBSPKSv2	PT	PKS/NRPS (structure/ substrate/ product)	2017	nii.ac.in/sbspks2.html	[103,104]
SEMPI 2	PT	genome-based PKS I modular product prediction; web-application	2017	pharmaceutical-bioinformatics.de/SeMPI/	[105]
CASSIS and SMIPS	PT	Promoter base- BGC prediction focused around SM-key enzymes, online or as application	2016	sbi.hki-jena.de/cassis	[106]
FunGene-ClusterS	PT	Transcriptome and genome-assisted BGC prediction; standalone and web application	2016	fungiminions.shinyapps.io/FunGeneClusterS	[107,108]
2metDB/SecmetDB	PT	PKS/NRPS-prediction tool; software application	2015	sourceforge.net/projects/secmetdb/	[109]
GNP	PT	Structure prediction and LC-MS data peak identification; web-application	2015	magarveylab.ca/gnp/	[110]
Smiles2Mono-mers (s2m)	PT	predict monomers from polymeric structure; webserver and standalone	2015	bioinfo.lifl.fr/norine/smiles2monomers.jsp	[111]
ClusterFinder	PT	BGC detection via Pfam domain occurrence in genome and metagenomic data; software application and integrated into antiSMASH and IMG-ABC	2014	github.com/petercim/ClusterFinder	[82]
MIPS-CG	PT	Clusters of BGC-related Pfam-domains outside of syntenic blocks; web-application	2014	fung-metb.net/ (currently offline)	[47]
MIDDAS-M	PT	Motif-Independent transcriptome and genome-assisted BGC prediction	2013	133.242.13.217/MIDDAS-M (currently offline)	[86]
ClusterMine-360	DB	crowd-source, semi and auto-curated database of microbial NRPS and PKS	2013	clustermine360.ca/ (currently offline)	[112]
NRPS-predictor2	PT	predict bacterial and fungal NRPS adenylation domain substrate specificity; web-application	2011	nrps.informatik.uni-tuebingen.de/ (currently offline)	[113]
SMURF	PT	Rule-based-backbone gene search via HMMs in genome data; web-application	2010	jcvi.org/smurf/	[79]
CLUSEAN	PT	PKS/NRPS-prediction tool; software application	2009	bitbucket.org/tilmweber/clusean	[114]

Abbreviations: Database (DB), Prediction Tool (PT).

**Table 2:** Successful application of BGC induction by mimicking environmental conditions or by genetic manipulation of global regulators.

BGC Activation by Natural Triggers and Manipulation of Their Global Regulators			
Section 1: *Aspergillus* and *Penicillium*			
Trigger	Organism	Observation	Ref.
*ApyapA∆*	*Aspergillus parasiticus*	*ApyapA∆*: increased aflatoxin production	[173]
*csnE∆*	*Aspergillus nidulans*	*csnE∆*: production of orsellinic acid-related metabolites: orcinol, diorcinol, cordyol C, violaceol I and violaceol II; activation of DHMBA cluster	[181,182]
*mcRA∆* (TF)	*Aspergillus nidulans* *Aspergillus terreus* *Penicillium canescens*	*mcRA∆*: Upregulation of secondary metabolites in all three organisms *A. nidulans* dereplication strain: two new metabolites from the cichorine pathway, production of felinone A new compounds of other organisms not identified	[175]
*sumO∆*	*Aspergillus nidulans*	*sumO∆*: production of Asperthecin, decrease of austinol/dehydroaustinol and sterigmatocystin	[183]
Addditives (supernatant extract)	*Aspergillus nidulans* *Streptomyces rapamycinicus*	a novel a guanidine containing macrolide named polaramycin B, produced by *S. rapamycinicus*, is responsible for the derepression of the ors- cluster and its derivates in *A. nidulans*	[184]
*Additives*	*Penicillium citrinum*	Cultivation in presence of rare earth metals (i.e., scandium chloride) and isolation of three new peptide derivatives	[185]
C-source ∆/OE of *creA*	*Aspergillus flavus*	*creA∆* (Carbon catabolite repressor) Growth defects, impaired conidia production, increased amount of sclerotia, near abolishment of aflatoxin production, impaired virulence OE of *creA*: similar to wild type	[122]
C-source	*Aspergillus nidulans*	Carbon source dependent BGC expression	[186]
C-source *laeA∆*	*Penicillium expansum*	sucrose decreases both patulin and *laeA* levels and increases *creA* expression LaeA regulates patulin BGC, *laeA∆* results in less virulence	[144]
Cultivation (chemostat) N starvation	*Aspergillus nidulans*	Production of antiproliferative compounds sanghaspirodins A and B Convergence of two BGCs pathways (anthraquinone and orsellinic acid-derived)	[128]
Light Temperature *veA∆* *laeA∆*	*Aspergillus fumigatus*	Connection between temperature and light regulation of 11 BGCs Involvement of VeA at 37 °C and LaeA at 30 °C and 37 °C in BGC regulation	[157]
Light *velB∆* *veA∆*	*Aspergillus nidulans*	velvet complex VelB/VeA/LaeA regulates developmental regulation and secondary metabolism in a light-dependent manner. *velB∆* or *veA∆*: defects in sexual fruiting-body formation and secondary metabolite production	[138]
Light *laeA∆, veA∆, velB∆*	*Aspergillus ochraceus*	*laeA∆*/*veA∆*/*velB∆*: differential regulation of 66% of all BGC backbone genes (majority downregulated) drastic reduction of Ochratoxin A production	[136]
Light Velvet: *∆Apc.LaeA∆ Apc.VeA∆*	*Aspergillus pachycristatus*	*Apc.LaeA∆* or *Apc.VeA∆* reduce production of echinocandin B and sterigmatocystin Impact of deletions on aerial hyphae, pigmentation, development of conidiophores, conidial germination rate, and ascospore maturation	[134]
Media Mimicking of plant and rhizosphere environment Nitrogen, Metals, Amino acids	*Aspergillus terreus*	(independent) signals for terrain production: methionine, nitrogen limitation, iron starvation (=host rhizosphere) Global regulators AreA and AtfA essential for terrain production	[119]
Media composition Carbon source Amino acids CreA	*Aspergillus terreus*	discovery of isoflavipucine and dihydroisoflavipucine production only in the presence of certain amino acids, alkaline pH, and strictly repressed in the presence of glucose (CreA).	[167]
Media C-source	*Penicillium citrinum*	Increased production of citrinin on glucose compared to sucrose	[187]
Media HDACi DNMTi	*Aspergillus awamori*	Media: MEA, rice, nutrient, triptone soya agar HDACi: valproic acid, nicotinamide, trichostatin A DNMTi: 5-azacytidine Different metabolic profiles between media and epigenetic modifiers Nicotinamide was found to be the best epigenetic inducer Rice grains were the best medium for SM induction	[188]
Metals and Trace elements	*Aspergillus fumigatus*	Xanthocillin BGC-derived isocyanides help accumulate copper and exhibit antimicrobial activity	[166]
Metals and Trace elements	*Aspergillus fumigatus*	HapX and SreA, which oppositely regulate iron homeostasis also control an iron-dependent secondary metabolite network comprising the virulence factor hexadehydroastechrome (tryptophan-derived iron (III)-complex) which causes an iron starvation phenotype upon overexpression	[165]
Metals and Trace elements Oxidative stress *sidC∆*	*Aspergillus nidulans*	NRPS SidC involved in iron regulation (excess and starvation) Upregulated by oxidative stress *sidC∆*: bad iron utilization (reduced growth under iron-starvation and higher iron demand), delayed germination under iron-depleted conditions, higher sensitivity of conidia to oxidative stress, no cleistothecia formation	[169]
Metals and Trace elements	*Penicillium brasilianum*	CuSO4 and MnSO_4_ alter the SM-profile production of a series of cyclodepsipeptides repression of verruculogen biosynthesis	[163]
Metals and Trace elements	*Penicillium urticae*	eight metal ions tested manganese dependent conversion of 6-methylsylicylic acid to patulin	[164]
OE *laeA*	*Aspergillus nidulans*	overexpression of *laeA*: activation of the Terrequinone (tdi) cluster and production of terrequinone A	[189]
Osmotic & Saline stress	*Aspergillus aculeatus* Three ex-type strains from three different collections	Osmotic (glycerol) and saline stress had different impacts on SM profile of fungal strains. Also strain-source specific chemotypes. Same morphology across all treatments	[159]
Oxidative stress Carbon source	*Aspergillus flavus*	Strains that produce less aflatoxin are more prone to oxidative stress and have more differentially regulated genes (also SM genes) under oxidative stress conditions carbon source affects BGC expression and production	[190]
Oxidative stress *ApyapA∆*	*Aspergillus ochraceus*	*ApyapA∆* shows missing redox balance which triggers aflatoxin synthesis and an involvement of oxidative stress in ochratoxin A regulation	[174]
Oxidative stress *veA∆*	*Aspergillus parasiticus*	transcription factor AtfB regulates conidial tolerance towards oxidative stress AtfB binds to promoters with CRE sites in the aflatoxin cluster under inducing conditions. *veA∆* abolishes these interactions	[171]
pH	*A. nidulans* *A. parasiticus*	Sterigmatocystin/aflatoxin expressed at higher levels at acidic pH than on neutral or alkalic pH Constitutive active PacC in *A. nidulans* reduced sterigmatocystin production	[150]
pH Acidic stress	Acid-Tolerant Fungi *Penicillium* *Aspergillus* *Talaromyces* *Cladosporium* *Allophoma* *Alternaria* *Trichoderma*	Supernatant extracts: ¾ shows cytotoxic activity, <¼ show antimicrobial or anti-H1N1 activity pH-related chemical diversity of *P. oxalicum*	[149]
pH *AcpacC∆*	*Aspergillus carbonarius*	PacC involved in pathogenicity and Ochratoxin A production *AcpacC∆*: impaired fungal growth at neutral/alkaline pH, diminished gluconic and citric acid production, reduced virulence toward grapes and nectarine fruits	[148]
pH Light Temperature	*Aspergillus flavus*	Aflatoxin production: 5x increase of in darkness pH 4 media produces 30 times more than pH 7.4 strongly reduced production at 18 or 40 °C	[191]
pH *pacC*	*Aspergillus nidulans*	Penicillin highest at alkaline pH and in mutated *pacC* (mimics alkaline conditions) Production was lowest at acid pH and in strains that mimic acid pH	[151]
Solid-state vs. submerged fermentation Media	*Aspergillus oryzae*	Solid-state: Increase of coumarins and oxylipins, higher antimicrobial activities submerged: Terpenoids abundant	[178]
Solid-state vs. submerged fermentation	*Penicillium expansum*	Submerged fermentation: strongly increased production of polyketide metabolites (agonodepside B, rotiorin, verrucosidin, and ochrephilone) Solid-state fermentation: exclusive production of meroterpenoid compounds (andrastin A and C)	[177]
Temperature	*Aspergillus fumigatus*	Temperature affects stress tolerance, pigmentation, and trypacidin accumulation in conidia	[192]
Temperature	*Aspergillus fumigatus*	Endocrocin biosynthesis in spores is temperature-dependent and endocrine is connected to pathogenicity	[193]
Temperature	*Penicillium oxalicum* *P. citrinum*	Unique metabolite profile under temperature stress	[194]
Temperature Oxidative stress Culture conditions	*Aspergillus flavus*	Float culture method Stress conditions: aflatoxin is downregulated under stress secondary metabolites with antioxidant properties (e.g., kojic acid and imizoquins) were upregulated under stress	[118]
Velvet: *ΔlaeA* *laeA-OE*	*Aspergillus* spp.	*laeA∆*: abolishment of sterigmatocystin, penicillin, and lovastatin *laeA-OE*: increased penicillin and lovastatin production *laeA* expression is negatively regulated by AflR	[142]
*mpkA∆* (kinase knockout library)	*Aspergillus nidulans*	*mpkA∆*: activation of the pkf-cluster and production of aspernidine A	[195]
**Section 2: *Fusarium***			
**Trigger**	**Organism**	**Observation**	**Ref.**
∆/OE *lae1*	*Fusarium fujikuroi*	*Δlae1*: downregulation of unknown NRPS4 cluster *OE-lae1* induced beauvericin cluster and STC7-cluster (product not identified) generally LaeA has a substantial impact on many SMs	[147]
*∆areA*, *∆areB*	*Fusarium fujikuroi*	deletions have an impact on >50% of BGCs *∆areB*: upregulation of cryptic clusters PKS09, NRPS04, NRPS21	[127]
*∆areA*, *areB*	*Fusarium fujikuroi*	AreA and *AreB* are required for gibberellic acid production	[196]
*∆pacC*	*Fusarium fujikuroi*	*pacC* deletion de-represses bikaverin cluster-complementation with active *pacC* variant represses bikaverin cluster	[153]
Global regulator of development *Δcsm1* (*NsdD*)	*Fusarium fujikuroi*	*Δcsm1*: elevated conidiation in wt and also in non-sporulating *ΔveA* strain. Induction of BGC for bikaverin and fusarubins Impact on 19 of 47 BGCs	[176]
Light N-source *∆veA*, *∆laeA*	*Fusarium oxysporum*	velvet complex and nitrate metabolism intertwined *∆veA* or *∆laeA*: growth impaired on nitrate/nitrite *AREA* involved in chromatin accessibility of two velvet-regulated BGCs (beauvericin and siderophore ferricrocin)	[131]
Light N- source pH *∆areA*, *∆areB* *∆vel1*, *∆lae1* *∆pacC*	*Fusarium fujikuroi*	*∆areA*: reduces fusaric acid production *∆areB*: abolished fusaric acid production *∆vel1* and *∆lae1*: significant reduction of fusaric acid *∆pacC*: abolishment of fusaric acid cluster expression	[197]
Light *∆fgwc-1* *∆fgwc-2*	*Fusarium graminearum*	*∆fgwc-1*/*∆fgwc-2*: impaired carotenogenesis, photoreactivation and maturity of perithecia mutants produced more aurofusarin and trichothecenes independent from light and have derepressed conidiation during constant light	[198]
Light *∆vel1, ∆vel2, ∆lae1*	*Fusarium fujikuroi*	*∆ffvel1* and *∆fflae1*: velvet positively regulates gibberellic acid, fumonisins and fusarin C, velvet negatively regulates bikaverin	[141]
N-source pH Host infection	*Fusarium fujikuroi* *F. circinatum* *F. mangiferae* *F. oxysporum*	PKS19 cluster is induced on rice but not maize Gibberelic acid cluster only expressed in *F. fujikuroi* Different conditions for different metabolites necessary: fusarins: high nitrogen, bikaverin and fusarubins: acidic low nitrogen or alkaline low nitrogen, Fumonisin B1: acidic low nitrogen	[125]
N-source *∆areA*	*Fusarium graminearum*	asparagine was found to be a preferential nitrogen source. *∆areA* led to poor growth on NaNO_3_ Utilization of aspartic acid, histidine, isoleucine, leucine, threonine, tyrosine, and valine as nitrogen sources was shown to depend of a functional AREA. AREA is required for the production of deoxynivalenol (DON), zearalenone, and fusarielin H regardless of the medium	[129]
N-source *∆areA*	*Gibberella fujikuroi*	∆areA: significantly reduced gibberellin production	[199], [200]
N-source/starvation ∆ of N-pathway regulators	*Fusarium Fujikuroi*	Effect of N-source (glutamine, nitrate) and starvation in different mutants *ΔniaD, ΔnrtA, ΔnirA* and *ΔareA* expression of backbone genes of gibberellic acid and bikaverin BGC after 72 h in starvation in wt and all mutants except for gibberellic acid in *ΔareA*	[124]
pH *PAC1*	*Fusarium graminearum*	Pac1 negatively regulates trichothecene BGC; induction under acidic pH Δfgpac1: reduced development under neutral and alkaline pH, increased sensitivity to H_2_O_2_ and early onset of Tri expression and product synthesis	[155]
Temperature	*Fusarium langsethiae* *F. sporotrichioides*	Impact of temperature and time on spore amount and T-2, HT-2 toxin production	[201]
**Section 3: Other Species**			
**Trigger**	**Organism**	**Observation**	**Ref.**
Additives	*Colletotrichum gloeosporioides*	Triggering silent BGCs by natural dietry components: Grape skin and turmeric extracts resulted in 20 and 14 additional compounds	[202]
C-source	*Spicaria elegans*	starch vs. glucose novel spicochalasin A and five new aspochalasins (M–Q)	[203]
Co-cultivation Additives	*Isaria felina*	potassium bromide and cocultivation with *Aspergillus sulphureus:* discovery of Isariketide B and Oxirapentyn L	[204]
Cultivation optimization for SM production	*Phomopsis* sp. *Hant25*	Optimization of different media and cultivation parameters for overproduction of mycoepoxydiene Parameters: combination of stationary and agitated, solid support (cellulose paper disc), different medias, cultivation time	[179]
Light *∆lreA* (=*WC-1*)	*Alternaria alternata*	Opposite light regulation of mycotoxin and spore production spores decreased under light and production of alternariol increased two to three times altertoxin production was strictly dependent on light. ∆ white-collar 1 (*WC-1*) gene (*lreA*): derepression of spore formation in dark and in light. altertoxin formation strongly induced in the dark alternariol formation partially dependent on LreA still able to partially respond to blue light	[205]
Light Velvet *∆velB* *∆veA* *∆laeA*	*Cuetootiopsis microspora*	Velvet Complex involved in the development, stress tolerance, and secondary metabolism *∆velB*: hypersensitive to osmotic stress and congo red pestalotiollide B production requires *velB* and *laeA*. *veA* inhibits pestalotiollide B biosynthesis	[132]
Light Velvet Iron starvation	*Neurospora crassa*	Velvet controls development, secondary metabolism, and light-dependent carotenoid production Heterotrimeric velvet complex (VE-1/VE-2/LAE-1) suppresses the production of the siderophore coprogen under iron starvation	[133]
Light C-source *∆CRE1*	*Trichoderma reesei*	Intertwined regulation of light and carbon pathways CRE1- regulated cluster is responsible for light-dependent production of dihydrotrichotetronin Biosynthesis of the antibiotic paracelsin was influenced	[135]
Light *∆SUB1*	*Trichoderma reesei*	*SUB1* is light-regulated SUB1 affects growth and is required for female fertility SM is regulated by SUB1 in a light- and nutrient-dependent manner	[206]
Media Salts Cultivation type	*Nigrospora* sp. *MA75*	Discovery of 2,3-didehydro-19a-hydroxy-14-epicochlioquinone B, 6-O-desmethyldechlorogriseofulvin 6′-hydroxygriseofulvin Media: NaCl vs. NaI, static/solid vs. agitated/liquid, rice-based vs. yeast extract	[207]
Media HDACi	*Drechslera* sp.,	HDACi: SAHA, valproate, octanoylhydroxamic acid 2 new metabolites: chromanone and prenylhydroxybenzoic acid different metabolome between MEA and minimal media biotransformation of the inhibitors occurred	[208]
Media HDACi DNMTi	*Cladosporium resinae*	Media. Czapek-DOX, YES, PDA, starch, liquid vs. solid HDACi: SAHA DNMTi: 5-AZA Czapek yeast extract broth: more total secondary metabolites DNMTi and HDACi: expressions of silent genes	[209]
N-source *∆AcareA*	*Acremonium chrysogenum*	AcareA is required for nitrogen metabolism and cephalosporin production	[130]
*OE-laeA*	*Chaetomium globosum*	overexpression of *laeA*: activation of the chaetoglobosin gene cluster and production of chaetoglobosin Z	[145]
pH *ΔpacC* *pacC^c^*	*Trichoderma virens*	*ΔpacC*: decreased competitiveness against *Rhizoctonia solani* and *Sclerotium rolfsii* Constitutive PacC: wt like overgrowth of *R. solani*	[152]
Plant Hormones	*Arthrinium sacchari*	discovery of the polyketides kinetin, 6-benzylaminopurine, and forchlorfenuron	[210]
Salinity	47 marine fungi isolates	NaCl and KCl had growth-promoting effect on most marine fungi and 15% of isolates showed increased antifungal activity against Candida albicans, NaCl had impact on the metabolite profile	[160]
Salinity	*Spicaria elegans*	Different secondary metabolites on different saline conditions (3% and 10%) Four metabolites, only at 10% salinity: (2E,2’Z)-3,3’-(6,6’-dihydroxybiphenyl-3,3’-diyl) (new compound), diacrylic acid, aspulvinone E, aspochalasin E and trichodermamide B	[162]
Solid vs. liquid fermentation Solid growth support	*Cylindrocarpon* sp. *Acremonium* sp. *Penicillium* sp.	Addition of inert absorbent polymeric attachment supports in solid state and submerged fermentation leading to distinct metabolite profiles Impact on culture morphology and relative metabolite yields Solid agar with moist polyester-cellulose paper: *Cylindrocarpon* sp. (pyrrocidines A and B), *Acremonium* sp. (acremonidins A–E) Agitated submerged cultures: Complex HPLC metabolome, decrease in target compounds Liquid fermentation with various inert growth supports (polypropylene, polypropylene cellulose, polyester-cellulose, or polyurethane): production of rugulosin, skyrin, flavomannin, and the new compound WF159-A (bisanthracene)	[180]
Temperature	40 polar fungal isolates	45% of fungal strains showed antimicrobial activity Different fungal metabolite profiles dependent on temperature (4, 10, 15, 28 °C)	[158]
*ΔvmlaeA*	*Valsa mali*	* ΔvmLaeA * : reduced virulence and differential regulated secondary metabolism profile (31/60).	[143]

The table is separated into three sections: Section 1 contains studies that were conducted on fungi of the genus Aspergillus or Penicillium, Section 2 comprises fungi from the genera Fusarium and Section 3 presents studies with filamentous fungi of other genera. The sections themselves are ordered alphabetically by the trigger that was applied. Signs and Abbreviations: Δ: deletion of a gene; OE: overexpression of a gene; TF: transcription factor.

**Table 3:** Discovery of new secondary metabolites by co-cultivation. Ordered by Set-up type.

BGC Activation by Co-Cultivation				
Setup	Method	Organism	Observation	Ref.
1	Co-cultivation of two developmental stages (morphs) of a marine alga-derived fungus Pre-grow both morphs on agar plates (2–3 weeks) → small liquid pre-cultures → combine mono-cultures after 14 days → cultivate for 15–20 days	*Aspergillus alliaceus* and its teleomorph *Petromyces alliaceus*	Production of the cytotoxic chlorinated bianthrone allianthrone A (only liquid) Morphs develop new phenotype in co-culture under liquid but not solid fermentation Restreaks after co-culture keep phenotype and can produce bianthrones on liquid and solid media	[218]
1	Submerse co-culture and membrane-separated culture 5 mL of pre-culture (A. flavipes and Streptomyces sp. in a ratio of 1:4 (*v*/*v*)) were added to 200 mL medium 8 days of cultivation	*Aspergillus flavipes* marine actinomycete *Streptomyces* sp.	Production of five aspochalasins and rosellichalasin by fungus with cytotoxic effects against Streptomyces. Physical interaction necessary	[219]
1	3 media (ISP2, GYE, F) inoculated with 3d old pre-cultures. 4L culture, fungal preculture was incubated 2 d earlier, 6 h before harvest 50 g/L Diaion HP-20 resin was added	*Aspergillus fumigatus* MR2012 (marine) *Streptomyces leeuwenhoekii* (C34 and C58) (desert)	Fungus: production of luteoride D (luteoride derivative), pseurotin G (pseurotin derivative), terezine D, 11-O-methylpseurotin A Bacteria C34: Chaxapeptin C58: Chaxapeptin doubled, pentalenic acid	[220]
1	16 h old mycelia of *A. fumigatus* were washed and placed in fresh AMM with 1/20 vol of the *streptomyces* culture or with 1/20 filtered supernatant. Harvest after 12 h	*Aspergillus fumigatus* *Streptomyces rapamycinicus*	induction of fungal PKS FgnA, the discovery of bacteria-specific germination inhibitor fumigermin	[221]
1	2 day 30 °C liquid agitated co-culture (93 mL London + 32 mL GYM medium). simultaneous inoculation of both MOs (equal amounts of spores)	*Aspergillus nidulans* *Streptomyces mobaraensis*	bacterial glycopeptide (antibiotic) triggers antibacterial and iron-chelating tropolonesanhydrosepedonin and antibiotic C (*A. nidulans*), polyketide tripyrnidone (novel structure)	[222]
1	Solid rice medium	*Aspergillus versicolor* *Bacillus subtilis*	two new tetralones; aspvanicin A and aspvanicin B several other cryptic SMs; cytotoxic activity against mouse lymphoma cells	[223]
1	Confrontation assay on ISP2 agar MALDI-TOF straight from agar Inoculation for 48 h at 30 °C	*Aspergillus fumigatus* *Aspergillus niger* *Bacillus* *amyloliquefaciens* (GA40)	The coral-derived bacteria GA40 produced an antifungal iturin	[224]
1	Precultures on rice media 1 week then co-culture for 2 weeks. Axenic cultivation with potassium bromide	*Aspergillus sulphureus* KMM 4640 *Isaria feline*	discovery of Isariketide B (rice media with KBr) and Oxirapentyn L (Co-cultivation)	[204]
1	Fungal bacterial stepwise liquid co-culture. Addition of bacterial broth (1/1000) to fungal culture on day 3. Further cultivation for 2 days	*Emericella* sp. (CNL-878; marine fungus) *Salinispora arenicola* (CNH-665; ascomycete)	Emericellamides A and B synthesis by *Emericella* sp.	[225]
1	Solid agar confrontation assay (5 days) Submerged fermentation (2 mL of each pre-culture and cultivation for 5 days in broth) Co-cultivation of MF028 with five other strains time-course	*Chaunopycnis* sp. (CMB-MF028) *Trichoderma hamatum* (CMB-MF030) Co-isolated from mollusc *Siphonaria* sp.	Chaunopyran A production Cultivation on sterilized mycelia showed no induction	[226]
1	Extraction of confrontation zone (Agar) Incubation of several weeks Dereplication via monocultures Method development: ~600 cultures by confrontation assay on agar UHPLC–TOF-MS fingerprints of mono and cocultures have been prepared to filter out new peaks	*Fusarium* isolates (clinical, soil or plant-derived) *Aspergillus clavatus, Trichophyton rubrum, Acremonium, Cladosporium* sp. *Hohenbuehelia reniformis* *Bionectria ochroleuca Eutypa lata*	Chemical novel MS spectra have been recorded Results indicate that a large portion of fungi produce new metabolites in co-culture	[213]
1	Solid state confrontation cultivation 17 basidiomycetes, 136 co-cultures Molecular network analysis Liquid co-cultivation for 5–30 days	*Ganoderma* *applanatum* *Trametes versicolor*	discovery of novel xylosides 3-phenyllactic acid and orsellinic acid in *G. applanatum*	[227]
1	Addition of bacterial broth (1/1000) to fungal culture on day 3. Harvest after 24 h. Screen of 50 fungal strains	*Libertella* sp. (CNL-523) *α-proteobacterium* (CNJ-328)	Discovery of Libertellenones A–D, produced by the addition of marine α-proteobacterium to a 3-day *Libertella* sp. culture.	[228]
1	Submerged fermentation Different pre-grow times before co-cultivation. Optimal: addition of fungal culture to 2-day bacterial culture and further incubation for 7 days. Other setups showed metabolome of either bacterial or fungal axenic culture	*Penicillium* sp. *(WC-29-5) Streptomyces fradiae* (007)	Production of five phenolic polyketides	[229]
1	Solid rice media, Cultivation parameters Optimal: inoculation of Penicillium sp mycelium plug. and seed medium of *Bacillus* sp. simultaneously at the center of petri dish 15d culture	*Penicillium* sp. (DT-F29) *Bacillus* sp. (B31)	Ten bioactive 2,5-diketopiperazines by *Penicillium* sp.	[230]
1	Co-cultivation on solid rice medium	*Penicillium strains* (IO1 and IO2) isolated from sponge	Accumulation of norlichexanthone and monocerin, biotransformation of the antifungal pyridoxatin to methyl-pyridoxatin	[231]
1	Liquid co-culture with seawater-based marine nutrient medium. After 24 h, bacterial culture is added to fungal cultivation following further 6d cultivation	*Pestalotia* (marine fungus) CNJ-328 (marine bacterium)	Discovery of pestaolne (antibiotic)	[216]
1	Submerged fermentation and sea water agar, inoculation of flasks with spores of both fungi, cultivation for 9 days	*Talaromyces aculeatus* *Penicillium variabile*	Four new polyketides (penitalarins A/ B/C and nafuredin B) cytotoxicity against some human cancer cell lines	[232]
2	Mycelia from *A nidulans* o/n preculture was combined with 1/20 (*v*/*v*) *Sreptomyces* preculture in fresh AMM medium. Further cultivation at 37 °C for 24 h	*Aspergillus nidulans* with a collection of 58 soil-dwelling actinomycetes	Physical interaction between the fungus and *Streptomyces hygroscopicus.* Induction of orsellinic acid and derivatives (lecanoric acid, yellow polyketides)	[215]
2	Co-culture in Warkingsman’s agar (Confrontation assay) and liquid medium (5 days)	*Fusarium graminearum* vs. 12.000 bacteria	reduced virulence of *F. graminearum* due to *Pseudomonas piscium* (FgGcn5 inhibitor phenazine-1-carboxamide)	[214]
3	treatment of *Aspergillus* with extracts of *Streptomyces* cultures	*Aspergillus nidulans* *Streptomyces rapamycinicus*	a guanidine containing macrolide, polaramycin B that is constitutively produced by the bacteria triggers the production of the ORS BGC	[184]
3	analytical scale microbial cultivations in a 24-well plate cultivation in presence of metabolites from other cultivation	*Aspergillus* sp. *Streptomyces* sp.	bacteriostatic cyclo-(L-Phe-trans-4-hydroxy-L-Pro) from *Aspergillus* stimulates nitric oxide production by *Streptomyces* which triggers silent BGC of *Aspergillus*: fungistatic heronapyrrole B	[233]

**Table 4:** Successful application of overexpression methods for induction of BGCs. Entries are ordered alphabetically by organism.

Targeted BGC Activation by Overexpression				
Method	Target/Parameter	Organism	Observation	Ref.
TF	*fsqA* (TF)	*Aspergillus fumigatus*	OE of *fsqA* (fsq gene cluster) production of pyrido [1,2-b]isoquinolines fumisoquin A and fumisoquin B	[249]
TF	*xanC*	*Aspergillus fumigatus*	OE of *xanC*: Activation of xan cluster and production of xanthocillin derivatives, the sulfated xanthocillin derivative BU-4704 and xanthocillin X monomethyl ether	[250]
CRISPRa	AN8506 AN8507 *mdpE*	*Aspergillus nidulans*	Method establishment: upregulation of putative BGC genes (AN8506, AN8507) and the silent mdP cluster via nucleosome map assisted targeting of inducible VPR-dCas9 into accessible chromatin in promoter	[251]
CRISPRa	*breF, fuml, fwnA*	*Aspergillus nidulans*	Method establishment: fusion of different chromatin active enzymes/domains to dCas9, activation of *breF* and *fuml* via p300-dCas9 (HAT), repression of *breF* by HAT GcnE-dCas9 and HDAC HosA-dCas9 and RpdA-dCas9 activation of *fwnA* by HosA-dCa9	[252]
CRISPRa (multiplex)	mic- BGC	*Aspergillus nidulans*	method establishment: activation of mic cluster by dCas12a-VPR (multigene CRISPRa): dehydromicroperfuranone production	[253]
Hybrid Activator	(+)-Asperlin cluster	*Aspergillus nidulans*	fusion of the DNA binding domain of AlnR (pathway-specific TF) and the trans-activation domain of AfoA lead to the activation of the (+)-Asperlin cluster	[246]
TF	aspyridone cluster (asp)	*Aspergillus nidulans*	overexpression of the pathway-specific TF *apdR*: activation of the silent BGC and production of aspyridone	[237]
TF	*ctnR*	*Aspergillus nidulans*	overexpression of the TF ctnR: Cluster activation (7 genes) and asperfuranone biosynthesis	[238]
TFBB gene	several BGCs	*Aspergillus nidulans*	Chicorine cluster activation pkg cluster activation: alternariol, citreoisocoumarin, analogs of citreoisocoumarin pki cluster: 6-Hydroxy-7-methyl-3-nonylisoquinoline-5,8-dione pkhB /pkhA: 2,4-dihydroxy-6-[(3E,5E,7E)-2-oxonona-3,5,7-trienyl]benzaldehyde Pkd: 2-ethyl-4,6-dihydroxy-3,5-dimethylbenzaldehyde	[243]
TF	*pbcR*	*Aspergillus nidulans*	overexpression of *pbcR* resulted in upregulation of a 7-gene cluster and the production of ent-pimara-8(14),15-diene	[254]
BB gene Het. Expr.	micro-perfuranone (micA, BGC)	*Aspergillus nidulans*	promoter exchange or heterologous expression (*A. niger*) of backbone gene *micA* was sufficient to produce microperfuranone	[255]
BB gene Het. Expr.	*asqK*	*Aspergillus nidulans*	OE of BB gene and heterologous expression for stepwise pathway elucidation: Products: 4′-methoxylated 6,7-benzodiazepinediones and 4′-methoxyviridicatin	[256]
Multiple genes	inpE cluster	*Aspergillus nidulans*	promoter replacement of seven genes (inpC-inpB): production of protease inhibitor fellutamide B	[248]
Multiple genes	ivo cluster	*Aspergillus nidulans*	OE of *ivoA*, *B* and *C* (and combinations): production of a dark pigment with the precursors N-acetyltryptophan and N-acetyl-6-hydroxytryptophan	[247]
Multiple genes	*sgdA, D, C, F*	*Aspergillus nidulans*	promoter replacements (4 genes): production of aspernidgulene A1 and A2	[257]
TF	*azaR* (TF)	*Aspergillus niger*	overexpression of *azaR*: Activation of aza cluster and production of azanigerones A–F	[258]
TF	*aflR*	*Aspergillus parasiticus*	overexpression of *aflR* lead to the production of aflatoxin under repressing conditions	[259]
TF	TF of pgm BGC	*Aspergillus terreus*	OE of pgm cluster TF (ATEG_06205) led to the production of naphthoquinones	[260]
Resistance gene	neomycin resistance	*Aspergillus versicolor*	The introduction of neomycin resistance leads to production of the new anti-tumor bioactive compounds	[261]
TF	NRPS31 BGC (APS-like) PKS19- BGC	*Fusarium fujikuroi*	overexpression of pathway-specific TF (*APS2*) and complementation of enzyme (*F. semitectum APS8*) resulted in production of ApicidinF OE of TF and PKS19 resulted in production of four novel compounds (Fujikurin A–D)	[125,262,263]
TF	*APF2*	*Fusarium fujikuroi*	overexpression of *APF2* increases apicidin F synthesis	[262]
Whole BGC	*BIK2* or *BIK3*	*Fusarium fujikuroi*	Separate OE of *BIK2* and *BIK3*: revelation of precursor oxo-pre-bikaverin	[264]
BB gene	*DMATS1*, FFUJ_09179	*Fusarium fujikuroi*	OE of *DMATS1* yielded a reversely N-prenylated tryptophan	[265]
TF	*TF22* (PKS-NRPS1)	*Fusarium fujikuroi*	Tet-on activation of *TF22* (FFUJ_02222): cluster activation and trichosetin production	[239]
TF	*FSL7* (TF)	*Fusarium graminearum*	overexpression of the TF *FSL7*: activation of the FSL cluster (*PKS9*) and production of fusarielins, F, G, and H	[266]
BB gene	*PKS8*	*Fusarium graminearum*	activation of Gibepyrones and Prolipyrone B production	[33]
TF	*FGM4* (fg3_54 BGC; NRPS)	*Fusarium graminearum*	OE triggers the production of fusaoctaxin A. Deletion compromises plant infection	[267]
BB gene	*FSDS*	*Fusarium heterosporum*	OE of backbone gene resulted in novel Tyrosine-Derived 2,4-Pyrrolidinedione	[268]
TF	*PEXANC* (TF)	*Penicillium expansum*	OE of *PEXANC* resulted in the activation of the Citrinin cluster via the TF CtnA and production of Citrinin (evolutionary TF repurposing) The theoretical cluster responsible for xanthocillin remained silent	[269]
Resistance gene	neomycin resistance	*Penicillium purpurogenum*	Introduction of neomycin resistance results in the production of the new cyclic dipeptide penicimutide	[270]
CRISPRa (TF)	*macR* (TF)	*Penicillium rubens*	induction of macrophorin pathway-specific TF *macR*: production of macrophorin A, D and 4′-oxomacrophorin D	[271]

Abbreviations: Heterologous expression (Het. Expr.), backbone gene (BB gene), pathway-specific Transcription factor (TF).

**Table 5:** Successful examples of heterologous expression of single genes and BGCs. Ordered by organism.

BGC Activation by Heterologous Expression			
Target/Parameter	Organisms	Observation	Ref.
high throughput BGC activation	22 Species (Donors) S. cerevisiae (Host)	method establishment: Hex Expression of 22/41 fungal BGCs resulted in detectable compounds	[285]
156 FACs with random genome fragments	*Aspergillus aculeatus* (Donor) *Aspergillus terreus* (Donor) *Aspergillus wentii* (Donor) *Aspergillus nidulans* (Host)	method establishment: FAC-MS, Identification of 15 new substances Clusters/metabolites: A. terreus: ATEG_07067, ATEG_07500, sesterterpenoid, valactamide A, benzomalvin A/D A. aculeatus: AACU_51108, AACU_59515, A. wentii: ASPWE_027400, ASPWE_034272, ASPWE_042595, ASPWE_044725, ASPWE_085322, ASPWE_151732, ASPWE_163793	[284]
diketomorpholine BGC	*Aspergilllus aculeatus* (Donor) *Aspergillus nidulans* (Host)	Elucidation of the biosynthetic pathway of acu-dioxomorpholine, featuring a new type of condensation domain, by FAC-MS	[287]
*ftmA* (NRPS)	*Aspergillus fumigatus* (Donor/Host) *Aspergillus nidulans* (Host)	OE of the NRPS *ftmA*: Production of Brevianamide F (Product of NRPS, product of cluster is putatively fumitremorgin)	[288]
pyr Cluster	*Aspergillus fumigatus* (Donor) *Aspergillus. oryzae* (Host)	successive expression of the pyr cluster: Characterization of pyr cluster and isolation of the meroterpenoid pyripyropene	[289]
micro-perfuranone (*micA*, BGC)	*Aspergillus nidulans* (Donor) *Aspergillus niger* (Host)	promoter exchange or heterologous expression of backbone gene *micA* was sufficient to produce microperfuranone	[255]
*asqJ* *asqE-L*	*Aspergillus nidulans* (Donor) *Escherichia coli* (Host)	OE of *asqE-L* in host and elucidation of AsqJ function by expression in *E. coli* Final products: 4′-methoxylated 6,7-benzodiazepinediones and 4′-methoxyviridicatin	[256]
*wA* (NWA)	*Aspergillus nidulans* (Donor) *Aspergillus oryzae* (Host)	expression of the WA-PKS: identification of the YWA1 compound	[290]
terreazepine BGC	*Aspergillus terreus* (Donor) *Aspergillus nidulans* (Host)	production of terreazepine	[291]
39 BGCs from various species	Donors: *Colletotrichum gloeosporioides* *Alternaria alternata* *Fusarium graminearum* *Trichoderma viride* *Aspergillus flavipes* Host: *Aspergillus oryzae*	method establishment: genome mining assisted automated and high-throughput (auto-HTP) biofoundry workflow refactoring of 39 BGCs and production of 185 distinct terpenoids	[292]
*STC5*, *STC3*	*Fusarium fujikuroi* (Donor) *E. coli* (Host)	Use of homologues *STC3*: (+)-eremophilene *STC5*:(¢)-guaia-6,10(14)-diene	[55]
carotenoid BGC of *Fusarium fujikuroi*	*Fusarium fujikuroi* (Donor) *Aspergillus nidulans* (Host)	method establishment: *aflR* scaffold as heterologous expression matrix; successful production of β-carotene	[286]
*STC1*	*Fusarium fujikuroi* (Donor) *E. coli* (Host)	Heterologous Expression of *STC1* identification of (–)-germacrene D	[176]
FgJ03939 BGC	*Fusarium graminearum* (Donor) *Saccharomyces cerevisiae* (Host)	stepwise synthesis of several new sesquiterpene synthase-derived metabolites such as fusariumdiene, fusagramineol	[293]
part of the fusarubin gene cluster	*Fusarium solani* (Donor) *S. cerevisiae* (Host)	Expression of *FVPPT*, fsr1, fsr2, and fsr3 in *S. cerevisiae* (galactose inducible promoter) and biosynthesis of bostrycoidin	[294]
*pvhA-E* (BGC)	*Penicillium variabile (Donor)* *Aspergillus nidulans (Host)*	Heterologous expression of genes *pvhA-e*: production of varicidin A and B	[295]
psilocybin BGC	*Psilocybe carpophores* (Donor) *Aspergillus nidulans* (Host)	method establishment: Successful Expression of whole BGC as polycistronic RNA piconavirus assisted expression of the whole basidiomycete derived psilocybin BGC	[296]
TESG_6702 to 6705	*Trichophyton tonsurans* (Donor) *Aspergillus nidulans* (Host)	Expression of the whole cluster resulted in the production of neosartoricins	[297]

**Table 6:** Untargeted BGC activation by interference with global chromatin regulatory mechanisms. Ordered alphabetically by organism.

BGC Activation by Interference of Chromatin-Regulation			
Method	Organism	Observation	Ref.
HDACi DNMTi	162 mangrove endophytic Fungi	HDACi: sodium butyrate, DNMTi: 5-azacytidine Activity against ESKAPE MOs and *Mycobacterium tuberculosis* and *Leishmania donovani*: 254/ 1608 extracts showed bioactivity, HDACi or DNMTi: 72 fungi produced active extracts, 40 fungi were active only in the control	[320]
Media HDACi DNMTi	*Aspergillus awamori*	Media: MEA, rice, nutrient, triptone soya agar HDACi: valproic acid, nicotinamide, trichostatin A, DNMTi: 5-azacytidine Different metabolic profiles between media and epigenetic modifiers Nicotinamide was found to be the best epigenetic inducer Rice grains was the best medium for SM induction	[188]
HDACi DNMTi	*Aspergillus calidoustus*, *Aspergillus westerdijkiae* *Aspergillus bombycis Aspergillus carbonarius* *Aspergillus fischeri*	HDACi: SAHA, DNMTi: 5-azacytidine significant changes in secondary metabolite profile with examples of both induction and repression.	[321]
HDACi DNMTi	*Aspergillus clavatus*	HDACi: valproic acid, trichostatin A, butyrate DNMTi: 5-azacytidine. N-acetyl-d-glucosamine Source of peptone strongly influences the SM profile, epigenetic modifier effects SM profile which is metabolite-specific and dependent on growth media and incubation time	[322]
Deletion	*Aspergillus nidulans*	*cclA∆* (Bre2; H3K4me): activation of mdp cluster: production of monodictyphenone, emodin, and emodin derivatives activation of anti-osteoporosis polyketides cluster: F9775A and F9775B	[323]
Deletion	*Aspergillus nidulans*	HepA occupancy decreases during sterigmatocystin activation *hepA∆*/*clrD∆* (H3K9me): upregulation of sterigmatocystin, isopenicillin A, terraquinone A LaeA involved in reversing heterochromatic marks at ST BGC,	[305]
Deletion	*Aspergillus nidulans*	*hosA∆* (HDAC): impact on H4ac, SM, carbohydrate metabolism, virulence, defense	[324]
Knockdown	*Aspergillus nidulans*	knockdown of *rpdA* (HDAC): production of aspercryptin A1 and A2 and fellutamides	[325,326]
HDACi	*Aspergillus niger*	HDACi: SAHA, discovery of nygerone A	[327]
Deletion	*Aspergillus niger*	*gcnE∆* (SAGA/Ada; acetylation): activation of 12 silent clusters (1 novel compound): nigerpyrone, carbonarone A, pestalamide A, funalenone, aurasperone E, aurasperone A	[328]
HDACi DNMTi	*Aspergillus* sp.	HDACi: suberohydroxamic acid, DNMTi: 5-azacytidine discovery of diphenylether-O-glycoside (diorcinol 3-O-α-D-ribofuranoside)	[329]
HDACi	*Botryosphaeria mamane*	HDACi: SAHA, Valporate, production of five unknown compounds	[330]
Deletion	*Calcarisporium arbuscula*	∆*hdaA* (HDAC): upregulation of 75% of BGC genes: isolation of 10 new compounds	[331]
HDACi DNMTi	*Chalara* sp. *6661*	HDACi: sodium butyrate, suberobishydroxamic acid, vorinostat; DNMTi: 5-azacytidine vorinostat: four new modified xanthones, only one out of four inhibitors showed effect Biotransformation of vorinostat	[332]
Media HDACi DNMTi	*Cladosporium resinae*	Media: Czapek-DOX, YES, PDA, starch, liquid vs. solid; HDACi: SAHA; DNMTi: 5-AZA Czapek, yeast extract broth: more total secondary metabolites DNMTi and HDACi: expressions of silent genes	[209]
HDACi DNMTi	*Cyathus stercoreus*	nine previously undescribed sesquiterpenes	[333]
Deletion	*Dothistroma septosporum*	*ΔDsKmt1* (H3K9me) or *ΔDsKmt6* (H3K27me): Substantial decrease of H3K9me3 or H3K27me3. Increase of production of dothistromin in both cases	[334]
Media HDACi	*Drechslera* sp.,	HDACi: SAHA, valproate, octanoylhydroxamic acid; 2 new metabolites: chromanone and prenylhydroxybenzoic acid; different metabolome between MEA and minimal media biotransformation of the inhibitors	[208]
Deletion	*Epichloe festucae*	*ΔclrD* (H3K9me) and/or *ΔezhB* (H3K27me): reduction of H3K9me3 and/or H3K27me3 activation of bioprotective lolitrems (ltm) and ergot alkaloids (eas)	[307]
Deletion	*Fusarium fujikuroi*	*Δhda1*(*hdaA*; HDAC): production of beauvericin (BEA-cluster; NRPS22)	[335]
Deletion	*Fusarium fujikuroi*	*Δset2* and *Δash1* (H3K36me): upregulation of PKS-NRPS9 and NRPS4	[336]
Deletion	*Fusarium fujikuroi*	∆*set1* (H3K4me): activation of PKSNRPS9, PKS type III, NRPS4, NRPS23, and DMATS3	[337]
Deletion	*Fusarium graminearum*	*Δkmt6* (PRC2, H3K27me): Differential regulation of many SM genes, upregulation of: NRPS9, NRPS14, NRPS16, NRPS19, PKS2, PKS3, PKS13, PKS22, PKS28, PKS29, STC5	[312]
Deletion	*Fusarium graminearum* (dereplication strain)	∆*kmt6* (H3K27me): discovery of Protofusarin and N-ethyl anthranilic acid, N-phenethylacetamide, N-acetyltryptamine ∆kmt6 and ∆fus1: tricinolone, tricinolonic acid	[338]
Deletion	*Fusarium mangiferae*	∆*fmkmt1* (H3K9me): increased beauvericin biosynthesis, nearly abolished fusapyrone production; revelation of fusapyrone BGC (FmPKS40)	[308]
Deletion	*Fusarium verticillioides*	*Δfvdim5* (H3K9me): increase of fumonisin B1 and melanin biosynthesis, reduced virulence	[339]
Deletion	*Metarhizium robertsii*	*Δhat1*: activation of 11 orphan BGCs Meromuside A–I, Meromutides A and B	[340]
HDACi DNMTi	*Muscodor yucatanensis*	HDACi: SAHA, DNMTi: 5-azacytidine different morphology, cultural characteristics, metabolites	[341]
HDACi DNMTi	*Nigrospora sphaerica*	HDACi: SAHA, sodium butyrate, valproic acid, quercetin DNMTi: 5-azacytidine, hydralazine hydrochloride induction of cryptic metabolites by all modifiers used	[342]
HDACi	*Penicillium brevicompactum*	HDACi: nicotinamide, sodium butyrate total phenolic compounds increased drastically upon HDACi treatment Nicotinamide: p-anisic acid, p-anisic acid methyl ester, benzyl anisate, syringic acid, sinapic acid, acetosyringone, phenyl acetic acid, gentisaldehyde and p-hydroxy benzaldehyde Sodium butyrate enhanced the productivity of anthranilic acid and ergosterol peroxide	[343]
Deletion	*Penicillium chrysogenum*	∆*hdaA* (HDAC): new compound. Maybe crosstalk with sorbicillinoids cluster	[344]
HDACi	*Phoma* sp.	SAHA triggers the production of (10’S)-verruculide B, vermistatin and dihydrovermistatin Absence of SAHA: discovery of (S,Z)-5-(3′,4′-dihydroxybutyldiene)-3-propylfuran-2(5H)-one	[345]
HDACi	*Trichoderma harzianum*	HDACi: sodium butyrate; diterpenoids changed into cyclonerane sesquiterpenoids. 3 new compounds: cleistanthane diterpenoid, harzianolic acid A, harziane diterpenoid, harzianone E, cyclonerane sesquiterpenoid, 3,7,11-trihydroxy-cycloneran	[346]
Knockdown	*Fusarium fujikuroi*	Knockdown of *KMT6* (H3K27me; PRC2): differential regulation of SM-clusters, upregulated SM genes: *NRPS22*, *STC4*, tetraterpene cyclase-encoding gene *TETC1*, *PKS-NRPS1, STC5, STC8, DMATS3, NRPS4, PKS2*) detection of six yet unidentified cluster-products: STC5: guaia-6,10(14)-diene, STC3: (+)-eremophilene	[55,315]

Abbreviations: HDACi (inhibition of histone deacetylases by chemical inhibitors), DNMTi (inhibition of DNA methyltransferases by chemical inhibitors), SAHA (vorinostat), 5-AZA (azacitidine).

## Data Availability

Not applicable.
